# GenAI-Empowered Network Evolution: Performance Analysis of AF and DF Relaying Systems over Dual-Hop Wireless Networks Under κ-μ Fading Case Study

**DOI:** 10.3390/s26041186

**Published:** 2026-02-11

**Authors:** Nenad Petrovic, Vuk Vujovic, Suad Suljovic, Milan Jovic, Dejan Milić

**Affiliations:** 1 Faculty of Electronic Engineering, University of Nis, 18104 Nis, Serbia; dejan.milic@elfak.ni.ac.rs; 2 Faculty of Information Technologies, MB University of Belgrade, 11040 Belgrade, Serbia; vuk.vujovic@ppf.edu.rs (V.V.); suadsara@gmail.com (S.S.); 3 The Academy of Applied Studies Polytechnic, Electrical and Computer Engineering, 11020 Belgrade, Serbia; mjovic@politehnika.edu.rs

**Keywords:** κ-µ fading, outage probability, average bit error rate, Binary Phase Shift Keying, Generative Artificial Intelligence (GenAI), Quadrature Phase Shift Keying

## Abstract

In this paper, the performance of dual-hop relay transmission in modern wireless communication systems is analyzed by considering two fundamental relaying techniques, namely, Amplify-and-Forward (AF) and Decode-and-Forward (DF). The propagation conditions on the source–relay (S-R) and relay–destination (R-D) links are modeled using the κ-μ statistical distribution, which effectively captures the fading characteristics in both line-of-sight (LoS) and non-line-of-sight (NLoS) environments. The analysis focuses on key performance metrics, including the outage probability (*P*_out_) and average bit error probability (*P*_e_), for Binary Phase Shift Keying (BPSK) and Quadrature Phase Shift Keying (QPSK) modulation schemes, assuming transmission via a single relay without a direct S–D link. Closed-form expressions for the considered metrics are derived based on the κ-μ model and verified by numerical evaluation. In addition to classical analytical modeling, a Generative Artificial Intelligence (GenAI)-enabled workflow is incorporated as a supportive tool in order to aid in automated analysis, the interpretation of the results in the context of network management under varying channel and system parameters based on the *P*_out_ and *P*_e_ calculations with the aim to tackle the underlying complexity and cognitive load of infrastructure adaptation and re-configuration operations. The combined analytical and GenAI-assisted approach provides valuable insights for the optimization, design, and continuous evolution of robust relay-based architectures in next-generation wireless networks.

## 1. Introduction

The evolution of modern wireless telecommunication systems has been largely driven by the growing demand for reliable, efficient, and seamless real-time information transmission. In practical wireless environments, the transmission of digitally modulated signals is subject to various degradation effects, among which fading plays a central role. Fading represents a stochastic fluctuation of the signal amplitude caused by reflections, scattering, and diffraction in the radio environment [1]. With the continuous increase in the number of users and the expansion of communication services, there is a critical need for the accurate modeling of wireless channels, including not only fading but also shadowing and inter-channel interference (ICI) [2]. Fast fading arises from multipath propagation, where multiple replicas of the signal with different amplitudes and phases interfere, while ICI originates from adjacent channel detection, particularly in dense frequency reuse scenarios [3]. Additionally, shadowing effects induced by urban infrastructure or dense vegetation reduce the line-of-sight (LoS) visibility and further degrade the link quality [4].

Due to the spectrum scarcity, frequency reuse has become a standard mechanism for improving capacity [5]. However, as the number of users grows, ICI often emerges as the dominant source of performance degradation [6]. To mitigate these limitations, relay-based transmission systems have been proposed, providing extended coverage and enhanced reliability without significant increases in transmit power or costly infrastructure expansion [7]. Relay systems are generally divided into two categories, non-regenerative (Amplify-and-Forward, AF) and regenerative (Decode-and-Forward, DF), each offering specific trade-offs in terms of complexity and performance [5,6].

In non-regenerative relay systems, the AF technique is employed, where the relay amplifies and retransmits the received signal without decoding. This approach simplifies implementation, reduces computational complexity, and enables low-latency operation, making it suitable for energy-efficient IoT networks, sensor systems, and next-generation 5G/6G communications [6,7]. In multi-hop AF systems, transmission occurs in two time slots: the source transmits to the relay in the first, while the relay forwards the amplified signal to the destination in the second. AF relays are generally classified as fixed-gain (blind) or channel state information (CSI)-based, with gain adapted to instantaneous channel conditions. While AF amplifies both signal and noise, it remains efficient when a trade-off between complexity and performance is required [7].

In regenerative relay systems, the DF technique is applied, where the relay decodes, optionally corrects errors, and retransmits the signal. DF enables error correction and improves system performance but requires more processing energy and depends on channel quality [8]. Despite the higher processing demands, DF systems can reduce the retransmissions and outage probability (*P*_out_), enhancing overall energy efficiency [9]. Modern designs also incorporate energy-harvesting (EH) relays, which exploit solar, Radio Frequency (RF), or vibration energy to extend the network lifetime without frequent battery replacement [10,11].

The introduction of relay nodes into a wireless system incurs additional energy consumption, as relays require power for transmission, reception, processing, and possibly signal re-encoding. In AF systems, energy is mainly consumed during amplification, while DF systems demand extra processing for decoding and re-encoding, increasing processor energy usage [8]. However, DF’s improved performance and lower outage probability and reduced average bit error can decrease retransmissions, further enhancing energy efficiency [9]. The relay transmit power is often set by a constant *c*, where *c* > 1 indicates reduced output power, allowing energy savings at the cost of some signal degradation. Modern approaches incorporate EH relays using ambient energy sources, enabling a longer operation without frequent battery replacement [10,11]. Overall, DF systems, despite higher processing demands, can reduce retransmissions and improve system stability, highlighting the importance of jointly analyzing reliability (*P*_out_, *P*_e_) and energy efficiency when designing relay networks [11].

For the accurate modeling of complex fading scenarios, where a line-of-sight (LoS) component is present alongside scattered components, the κ-μ distribution is used [12,13]. The parameter κ represents the ratio of the power of the dominant component to the total power of the scattered components, while μ denotes the number of multipath clusters and the severity of fading. This model encompasses Nakagami-*m* (κ = 0) and Rician (μ = 1) distributions, including Rayleigh and Gaussian distributions, providing a flexible and physically meaningful parameterization [13]. Understanding the dynamics of the κ-μ channels through modeling and simulations is crucial for the design and optimization of next-generation wireless systems, as it enables the accurate prediction of performance under diverse propagation conditions. The κ-μ model is particularly suitable for channels with a dominant LoS component and multiple scattering clusters, such as vehicular networks, Unmanned Aerial Vehicle (UAV) links, and microwave/millimeter-wave communications [14,15]. It is also successfully applied in indoor small-cell environments, where multipath propagation arises from multiple dominant reflected clusters [16]. Since the κ-μ distribution includes Rayleigh, Rice, and Nakagami-*m* as special cases, it provides a general framework suitable for the performance analysis of relay-based wireless systems under various propagation scenarios [14].

This paper investigates the performance of AF and DF relay systems operating in κ-μ fading channels, with a focus on the *P*_out_ and average bit error probability (*P*_e_) for digital modulation schemes, specifically BPSK and QPSK [17]. BPSK transmits one bit per symbol using two carrier phases (0° and 180°), whereas QPSK conveys two bits per symbol across four phases (0°, 90°, 180°, and 270°), effectively doubling the spectral efficiency while maintaining a comparable detection complexity [18]. Evaluating these modulation schemes under κ-μ fading conditions is crucial for designing robust, high-capacity, and energy-efficient wireless networks, particularly in environments with severe fading and multipath propagation [17,18]. The study emphasizes the influence of relay strategies and modulation selection on system performance, thereby supporting the development of reliable next-generation wireless communication systems. The analytical derivation in this paper is carried out for BPSK and QPSK modulation schemes, which allow closed-form expressions and a clear performance interpretation. Although the framework can be extended to higher-order modulations such as M-QAM, this would result in significantly more complex analytical expressions and is therefore beyond the scope of this work.

On the other side, recent advances in Generative Artificial Intelligence (GenAI) have introduced a range of complementary paradigms that support automation, reasoning, and knowledge extraction in complex engineering systems. Large Language Models (LLMs) provide strong capabilities for understanding, analyzing, and generating technical content, enabling the efficient handling of mathematical models, analytical procedures, and performance evaluation workflows. Vision–Language Models (VLMs) further extend these capabilities by incorporating visual inputs, such as system block diagrams, relay configurations, and performance plots, which are common in wireless communications analysis. In parallel, Retrieval-Augmented Generation (RAG) improves efficiency, consistency, and reliability by grounding model outputs in relevant external knowledge, such as standardization-related documents, reference architecture, requirements, and analytical results in order to reduce manual effort needed for providing the right context to the LLM prompts. Building on these paradigms, this paper also proposes a GenAI-empowered supportive framework that considers network management based on the performance analysis of dual-hop wireless networks employing amplify-and-forward and decode-and-forward relaying under κ-μ fading conditions as a case study. We leverage GenAI to aggregate information about the network and its current state, identify relevant events and anomalies, support infrastructure management decisions, and generate corresponding commands that will be applied to the target infrastructure in order to respond to the performance drops, topology, or environment changes. By combining classical analytical modeling with GenAI-enabled workflows, this approach aims to support the continuous evolution of the network under topology changes and varying environment conditions, demonstrating how LLMs, VLMs, and RAG can assist the analysis and analysis of relay-based wireless network deployments. Compared to the existing solution, we target greater extendibility and flexibility with the help of GenAI, while keeping the explainability and verifiability as a result of the elements of formal representations and methods incorporated from model-driven engineering (MDE).

The remainder of the paper is organized as follows. Section 2 presents the analytical derivation of the system outage probability and average bit error probability for the AF relaying technique, including the performance evaluation for QPSK and BPSK modulation schemes. Section 3 extends the analysis to the DF technique, providing outage probability and error rate results under varying channel parameters. Section 4 is devoted to numerical analysis and a comparative evaluation of AF and DF relaying techniques under κ-μ fading conditions. Section 5 introduces a dedicated GenAI-empowered framework for fading-aware network maintenance and automation, including related works and motivation, and presents an evaluation of both locally deployed and commercial GenAI models within the proposed workflow. Finally, Section 6 concludes the paper by summarizing the main contributions and outlining the future research directions. The study uses the same topology with a single source, relay, and destination, considering the case without a direct S–D component in the κ-μ channel. The comparison between AF and DF schemes is based solely on numerical results under identical channel and system conditions, allowing a fair assessment of their relative performance in terms of outage probability and error probability, without introducing additional system complexity.

## 2. System Outage Probability and Average Bit Error Probability for the AF Technique

**Figure 1:**
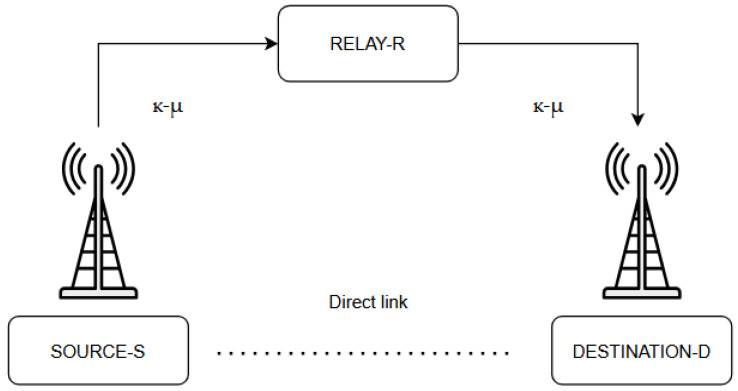
Relay transmission model under κ-μ fading on both links.

This chapter examines the transmission of a digitally modulated signal in a single-relay wireless system under κ-μ fading, employing the AF protocol. The system, depicted in Figure 1, consists of a source (S), a relay (R), and a destination (D), with the direct S–D link neglected. Fading is described by parameters κ_1_, μ_1_, and γ_1_ for the S–R link and κ_2_, μ_2_, and γ_2_ for the R–D link, where γ_1_ and γ_2_ represent the instantaneous signal-to-noise ratio (SNR) values on the respective links. AF is preferred for its simplicity: the relay amplifies the received signal with gain G and forwards it without decoding, reducing the hardware complexity and latency compared to DF systems. Although noise is also amplified, AF remains effective in severely degraded channels. When the channel state information (CSI) is available, G can be adjusted dynamically; otherwise, a fixed-gain (blind AF) with the mathematically equivalent G = 1 is applied. The Outage Probability (*P*_out_) and Average Bit Error Probability (*P*_e_) are computed numerically, assuming symmetric parameters (μ_1_ = μ_2_ = μ, κ_1_ = κ_2_ = κ, γ_th1_ = γ_th2_ = γ_th_), enabling the assessment of channel effects and offering guidance for the robust relay design in next-generation wireless networks.

### 2.1. System Outage Probability for the AF Technique

For a precise performance analysis of the relay system, it is essential to determine the statistical characteristics of the received signal, particularly its Probability Density Function (PDF) and Cumulative Distribution Function (CDF). These functions describe the behavior of the signal transmitted over the source–relay (S–R) and relay–destination (R–D) links, with the propagation environment modeled by the κ-μ distribution. The PDF of the received SNR, subject to κ-μ fading on any of the considered links, can be analytically expressed as [19], Equation (2.6):

(1) $$p_{\gamma }\left(\gamma \right)=e^{-\mu \kappa -\frac{\mu \left(1+\kappa \right)}{w}\gamma }\overset{+\infty }{\underset{i_{1}=0}{\sum }}\frac{\mu^{2i_{1}+\mu }\kappa^{i_{1}}\gamma^{i_{1}+\mu -1}}{\Gamma \left(i_{1}+\mu \right)i_{1}!}{\left(\frac{1+\kappa }{w}\right)}^{i_{1}+\mu }.$$

where Γ(·) is the Gamma function, parameter κ represents the ratio between the total power of the dominant component and the total power of the scattered components, μ denotes the number of multipath clusters and the fading severity, γ is the instantaneous signal-to-noise ratio, and *w* denotes the average SNR.

Using the definition for the Cumulative Distribution Function (CDF) of the instantaneous SNR for the κ-μ distribution [20]:

(2) $$F_{\gamma }\left(\gamma \right)=\overset{\gamma }{\underset{0}{\int }}p\left(t\right)dt.$$

we obtain CDF of the SNR:

(3) $$F_{\gamma }\left(\gamma \right)=\frac{1}{e^{\mu \kappa }}\overset{+\infty }{\underset{i_{1}=0}{\sum }}\frac{{\left(\mu \kappa \right)}^{i_{1}}}{\Gamma \left(i_{1}+\mu \right)i_{1}!}\overset{\frac{\mu \left(1+\kappa \right)}{w}\gamma }{\underset{0}{\int }}s^{i_{1}+\mu -1}e^{-s}ds.$$

The integral in (3) is evaluated using the identity [21], Equation (11.1.9):

(4) $$\overset{c}{\underset{0}{\int }}x^{b-1}e^{-x}dx=\gamma \left(b,c\right).$$

where γ(*b*, *c*) is the lower Gamma function, which can be expressed in terms of the Gamma function Γ(·) [22], Equations (8.352.6) and (8.339.1) as:

(5) $$\gamma \left(n,x\right)=\Gamma \left(n\right)-\Gamma \left(n\right)e^{-x}\overset{n-1}{\underset{i=0}{\sum }}\frac{x^{i}}{i!}=\Gamma \left(n\right)\left(1-e^{-x}\overset{n-1}{\underset{i=0}{\sum }}\frac{x^{i}}{i!}\right).$$

By applying expressions (4) and (5) into expression (3), the CDF of the SNR under the κ-μ distribution becomes:

(6) $$F_{\gamma }\left(\gamma \right)=\frac{1}{e^{\mu \kappa }}\overset{+\infty }{\underset{i_{1}=0}{\sum }}\frac{{\left(\mu \kappa \right)}^{i_{1}}}{i_{1}!}\left(1-e^{-\frac{\mu \left(1+\kappa \right)}{w}\gamma }\overset{i_{1}+\mu -1}{\underset{i_{2}=0}{\sum }}\frac{1}{i_{2}!}{\left(\frac{\mu \left(1+\kappa \right)}{w}\gamma \right)}^{i_{2}}\right).$$

The system outage probability is defined as the probability that the observed SNR at the receiver falls below a predetermined threshold γ_th_, which is set according to the Quality of Service (QoS) standards at the receiver [23], Equation (4.4):

(7) $$P_{out}=F_{\gamma_{eq}}(\gamma_{th})=1-\int_{0}^{+\infty }C_{\gamma_{1}}\left[\gamma_{th}+\frac{{\gamma_{th}}^{2}+c\gamma_{th}}{t}\right]p_{\gamma_{2}}\left[\gamma_{th}+t\right]dt.$$

where Cγ*_i_*(·) is the complementary CDF of γ*_i_* [20], Equation (1.5):

(8) $$C_{\gamma_{i}}(\cdot )=|1-F_{\gamma_{i}}(\cdot )|.$$

By substituting expression (6) into expression (8), we obtain the complementary cumulative function Cγ_1_(x) of the SNR CDF for the S–R link under the κ-μ distribution:

(9) $$C_{\gamma_{1}}(x)=C_{\gamma_{1}}\left(\gamma_{th}+\frac{{\gamma_{th}}^{2}+c\gamma_{th}}{t}\right)=1-F_{\gamma_{1}}\left(\gamma_{th}+\frac{{\gamma_{th}}^{2}+c\gamma_{th}}{t}\right)=1-\frac{1}{e^{\mu \kappa }}\cdot \;\cdot \overset{+\infty }{\underset{i_{1}=0}{\sum }}\frac{{\left(\mu \kappa \right)}^{i_{1}}}{i_{1}!}\left(1-e^{-\frac{\mu \left(1+\kappa \right)}{w}\left(\gamma_{th}+\frac{{\gamma_{th}}^{2}+c\gamma_{th}}{t}\right)}\overset{i_{1}+\mu -1}{\underset{i_{2}=0}{\sum }}\frac{1}{i_{2}!}{\left(\frac{\mu \left(1+\kappa \right)}{w}\left(\gamma_{th}+\frac{{\gamma_{th}}^{2}+c\gamma_{th}}{t}\right)\right)}^{i_{2}}\right).$$

where *c* ∈{0,1} is a binary indicator that distinguishes between AF relaying with a fixed gain (*c* = 0) and CSI-assisted (Channel State Information, CSI) AF relaying with an adaptive gain (*c* = 1), and γ_th_ is the threshold indicating the probability that the received SNR falls below the predetermined value.

Using (1), the SNR PDF at the receiver for the S–R–D link is:

(10) $$p_{\gamma_{2}}\left(\gamma_{th}+t\right)=\frac{1}{e^{\mu \kappa }}\overset{+\infty }{\underset{i_{3}=0}{\sum }}\frac{\mu^{2i_{3}+\mu }\kappa^{i_{3}}{\left(\gamma_{th}+t\right)}^{i_{3}+\mu -1}}{\Gamma \left(i_{3}+\mu \right)i_{3}!}{\left(\frac{1+\kappa }{w}\right)}^{i_{3}+\mu }e^{-\frac{\mu \left(1+\kappa \right)}{w}\left(\gamma_{th}+t\right)}.$$

By substituting expressions (9) and (10) into expression (7), the system outage probability at the receiver D for the S–R–D link is:

(11) $$P_{out}=1-e^{-\mu \kappa -\frac{\mu \left(1+\kappa \right)}{w}\gamma_{th}}\overset{+\infty }{\underset{i_{3}=0}{\sum }}\frac{\mu^{2i_{3}+\mu }\kappa^{i_{3}}}{i_{3}!\Gamma \left(i_{3}+\mu \right)}{\left(\frac{1+\kappa }{w}\right)}^{i_{3}+\mu }\left[\int_{0}^{+\infty }{\left(\gamma_{th}+t\right)}^{i_{3}+\mu -1}e^{-\frac{\mu \left(1+\kappa \right)}{w}t}\right.-\;-e^{-\mu k}\overset{+\infty }{\underset{i_{1}=0}{\sum }}\frac{{\left(\mu \kappa \right)}^{i_{1}}}{i_{1}!}\int_{0}^{+\infty }{\left(\gamma_{th}+t\right)}^{i_{3}+\mu -1}e^{-\frac{\mu \left(1+\kappa \right)}{w}t}+e^{-\mu k-\frac{\mu \left(1+k\right)}{w}\gamma_{th}}\overset{+\infty }{\underset{i_{1}=0}{\sum }}\overset{i_{1}+\mu -1}{\underset{i_{2}=0}{\sum }}\frac{\mu^{i_{1}+i_{2}}\kappa^{i_{1}}}{i_{1}!i_{2}!}\cdot \;\cdot {\left(\frac{\gamma_{th}\left(1+\kappa \right)}{w}\right)}^{i_{2}}\int_{0}^{+\infty }{\left(\gamma_{th}+t\right)}^{i_{3}+\mu -1}{\left(\gamma_{th}+c+t\right)}^{i_{2}}\frac{1}{t^{i_{2}}}e^{-\frac{\mu \left(1+\kappa \right)}{w}t}\left.e^{-\frac{\mu \gamma_{th}\left(1+k\right)\left(\gamma_{th}+c\right)}{w}\frac{1}{t}}\right].$$

Expression (11) involves the following integrals:

(12) $$I_{1}=\int_{0}^{+\infty }{\left(\gamma_{th}+t\right)}^{i_{3}+\mu -1}e^{-\frac{\mu \left(1+\kappa \right)}{w}t}dt\;I_{2}=\int_{0}^{+\infty }{\left(\gamma_{th}+t\right)}^{i_{3}+\mu -1}{\left(\gamma_{th}+c+t\right)}^{i_{2}}\frac{1}{t^{i_{2}}}e^{-\frac{\mu \left(1+\kappa \right)}{w}t}e^{-\frac{\mu \gamma_{th}\left(1+\kappa \right)\left(\gamma_{th}+c\right)}{w}\frac{1}{t}}dt.$$

Using the expression from [22], Equation (3.382.4):

(13) $$\int_{0}^{+\infty }{\left(\beta +x\right)}^{v}e^{-\mu x}dx=\mu^{-v-1}e^{\beta \mu }\Gamma \left(v+1,\beta \mu \right).$$

where Γ(α, *x*) is the lower or complementary incomplete Gamma function, which can be expressed using the formulae [22], Equations (8.352.4) and (8.339.1):

(14) $$\Gamma \left(n,x\right)=\left(n-1\right)!e^{-x}\overset{n-1}{\underset{k=0}{\sum }}\frac{x^{k}}{k!}=\Gamma \left(n\right)e^{-x}\overset{n-1}{\underset{k=0}{\sum }}\frac{x^{k}}{k!}.$$

The integral *I*_1_ can be written in the following form:

(15) $$I_{1}=\Gamma \left(i_{3}+\mu \right)e^{-\frac{\mu \left(1+k\right)}{w}\gamma_{th}}\overset{i_{3}+\mu -1}{\underset{i_{4}=0}{\sum }}\frac{{\gamma_{th}}^{i_{4}}}{i_{4}!}{\left(\frac{w}{\mu \left(1+\kappa \right)}\right)}^{i_{3}-i_{4}+\mu }.$$

Using the binomial theorem [22], Equation (1.111):

(16) $${\left(a+x\right)}^{n}=\overset{n}{\underset{k=0}{\sum }}\left(\begin{array}{c} n\\ k \end{array}\right)a^{n-k}x^{k}.$$

The integral *I*_2_ from expression (12) can be written as:

(17) $$I_{2}=\overset{i_{3}+\mu -1}{\underset{i_{5}=0}{\sum }}\overset{i_{2}}{\underset{i_{6}=0}{\sum }}\left(\begin{array}{c} i_{2}\\ i_{6} \end{array}\right)\left(\begin{array}{c} i_{3}+\mu -1\\ i_{5} \end{array}\right){\gamma_{th}}^{i_{3}-i_{5}+\mu -1}{\left(\gamma_{th}+c\right)}^{i_{2}-i_{6}}\int_{0}^{+\infty }t^{i_{5}+i_{6}-i_{2}}e^{-\frac{\mu \gamma_{th}\left(1+k\right)\left(\gamma_{th}+c\right)}{wt}-\frac{\mu \left(1+k\right)}{w}t}dt.$$

By applying the formula [22], Equation (3.471.9):

(18) $$\overset{+\infty }{\underset{0}{\int }}x^{v-1}e^{-\frac{\beta }{x}-\gamma x}dx=2{\left(\frac{\beta }{\gamma }\right)}^{\frac{v}{2}}K_{v}\left(2\sqrt{\beta \gamma }\right).$$

where *K*_v_(x) is the second-order Bessel function, the integral *I*_2_ can be expressed as:

(19) $$I_{2}=2\overset{i_{3}+\mu -1}{\underset{i_{5}=0}{\sum }}\overset{i_{2}}{\underset{i_{6}=0}{\sum }}{\gamma_{th}}^{\frac{2i_{3}+i_{6}-i_{2}-i_{5}+2\mu -1}{2}}{\left(\gamma_{th}+c\right)}^{\frac{i_{2}+i_{5}-i_{6}+1}{2}}\left(\begin{array}{c} i_{2}\\ i_{6} \end{array}\right)\cdot \;\cdot \left(\begin{array}{c} i_{3}+\mu -1\\ i_{5} \end{array}\right)K_{i_{5}+i_{6}-i_{2}+1}\left(\frac{2\mu \left(1+\kappa \right)}{w}\sqrt{\gamma_{th}\left(\gamma_{th}+c\right)}\right).$$

Substituting (15) and (19) into (11) and simplifying yield the CDF of the instantaneous SNR at the receiver, representing the system outage probability under κ-μ fading:

(20) $$P_{out}=1-e^{-\mu \kappa -\frac{\mu \left(1+k\right)}{w}\gamma_{th}}\overset{+\infty }{\underset{i_{3}=0}{\sum }}\frac{\mu^{i_{3}}\kappa^{i_{3}}}{i_{3}!}\left[\overset{i_{3}+\mu -1}{\underset{i_{4}=0}{\sum }}\frac{\mu^{i_{4}}}{i_{4}!}{\left(\frac{\gamma_{th}\left(1+\kappa \right)}{w}\right)}^{i_{4}}\right.-e^{-\mu \kappa }\overset{+\infty }{\underset{i_{1}=0}{\sum }}\frac{{\left(\mu \kappa \right)}^{i_{1}}}{i_{1}!}\left(\overset{i_{3}+\mu -1}{\underset{i_{4}=0}{\sum }}\frac{\mu^{i_{4}}}{i_{4}!}\right.\cdot \;\cdot {\left(\frac{\gamma_{th}\left(1+\kappa \right)}{w}\right)}^{i_{4}}-2e^{-\frac{\mu \left(1+\kappa \right)}{w}\gamma_{th}}\overset{i_{1}+\mu -1}{\underset{i_{2}=0}{\sum }}\overset{i_{3}+\mu -1}{\underset{i_{5}=0}{\sum }}\overset{i_{2}}{\underset{i_{6}=0}{\sum }}\frac{\mu^{i_{2}+i_{3}+\mu }}{i_{2}!\Gamma \left(i_{3}+\mu \right)}{\left(\frac{1+\kappa }{w}\right)}^{i_{2}+i_{3}+\mu }\left(\begin{array}{c} i_{2}\\ i_{6} \end{array}\right)\left(\begin{array}{c} i_{3}+\mu -1\\ i_{5} \end{array}\right)\cdot \;\cdot {\gamma_{th}}^{\frac{i_{2}+2i_{3}+i_{6}-i_{5}+2\mu -1}{2}}{\left(\gamma_{th}+c\right)}^{\frac{i_{2}+i_{5}-i_{6}+1}{2}}\left.\left.K_{i_{5}+i_{6}-i_{2}+1}\left(\frac{2\mu \left(1+k\right)}{w}\sqrt{\gamma_{th}\left(\gamma_{th}+c\right)}\right)\right)\right].$$

**Figure 2:**
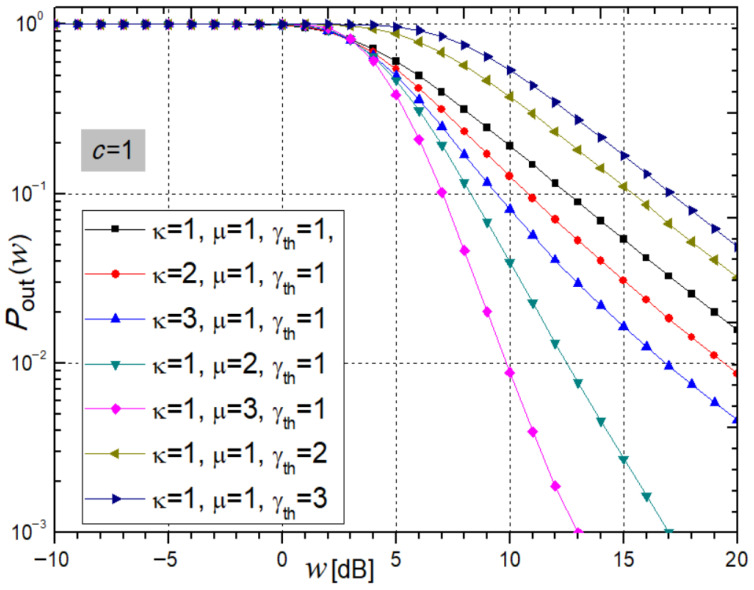
System outage probability from expression (20) for varying κ, μ, and γ_th_.

Figure 2 illustrates the receiver *P*_out_ from expression (20) versus the average SNR for varying κ, μ, and γ_th_.

Increasing the parameters κ and μ leads to a reduction in *P*_out_, thereby enhancing system stability. In contrast, an increase in the threshold parameter γ_th_ results in a notable rise in *P*_out_, indicating reduced stability and degraded system performance. For the parameter set κ = μ = γ_th_ = 2 at an average SNR of *w* = 5 dB, the system outage probabilities computed using Mathematica are 0.5453, 0.4677, and 0.8799, respectively.

Table 1 below shows the number of terms required to achieve an accuracy up to the fifth decimal place in the series representing *P*_out_ (20). For the given values of the system parameters, a maximum of 14 terms is sufficient to reach this level of accuracy when varying the parameters κ, μ, and γ_th_.

For an average SNR of *w* = −10 dB, the convergence of the series is achieved with just five terms for all values of the parameters κ, μ, and γ_th_. As γ_th_ increases, at *w* = 0 dB, convergence is reached with six terms, while, at *w* = 10 dB, more terms are required and the series converges more slowly. Similarly, when the parameters μ and κ increase, a larger number of terms is needed for *w* = 0 dB and *w* = 10 dB, resulting in a slower series convergence.

### 2.2. Average Bit Error Probability at the Receiver for the AF Technique

One of the key first-order statistical metrics is the Average Bit Error Rate (ABER), which characterizes the channel in terms of errors occurring during signal transmission. Based on the ABER, the performance of a wireless communication system employing relay-assisted transmission under κ-μ fading can be evaluated. The average bit error probability (*P*_e_) for modulation schemes such as QPSK (β = 1) and BPSK (β = 2) at the receiver can be expressed as [23], Equation (4.9):

(21) $$P_{e}=\frac{1}{\sqrt{2\pi }}\overset{+\infty }{\underset{0}{\int }}F_{\gamma_{th}}\left(\frac{t^{2}}{\beta }\right)e\left(-\;\frac{t^{2}}{2}\right)dt.$$

By applying expression (20) to (21), with γ_th_ = t^2^/β, the *P*_e_ can be expressed as:

(22) $$P_{e}=\frac{1}{\sqrt{2\pi }}\left[\overset{+\infty }{\underset{0}{\int }}e^{-\;\frac{t^{2}}{2}}dt\right.-e^{-\mu \kappa }\overset{+\infty }{\underset{i_{3}=0}{\sum }}\overset{i_{3}+\mu -1}{\underset{i_{4}=0}{\sum }}\frac{\mu^{i_{3}+i_{4}}\kappa^{i_{3}}}{i_{3}!i_{4}!}{\left(\frac{1+\kappa }{w}\right)}^{i_{4}}\overset{+\infty }{\underset{0}{\int }}{\left(\frac{t^{2}}{\beta }\right)}^{i_{4}}e^{-\;\frac{t^{2}}{2}-\frac{\mu \left(1+\kappa \right)}{w}\left(\frac{t^{2}}{\beta }\right)}dt+\;{+e}^{-2\mu \kappa }\overset{+\infty }{\underset{i_{1}=0}{\sum }}\overset{+\infty }{\underset{i_{3}=0}{\sum }}\overset{i_{3}+\mu -1}{\underset{i_{4}=0}{\sum }}\frac{\mu^{i_{1}+i_{3}+i_{4}}\kappa^{i_{1}+i_{3}}}{i_{1}!i_{3}!i_{4}!}{\left(\frac{1+\kappa }{w}\right)}^{i_{4}}\overset{+\infty }{\underset{0}{\int }}{\left(\frac{t^{2}}{\beta }\right)}^{i_{4}}e^{-\;\frac{t^{2}}{2}-\frac{\mu \left(1+\kappa \right)}{w}\left(\frac{t^{2}}{\beta }\right)}dt-2e^{-2\mu \kappa }\overset{+\infty }{\underset{i_{1}=0}{\sum }}\overset{i_{1}+\mu -1}{\underset{i_{2}=0}{\sum }}\;\overset{+\infty }{\underset{i_{3}=0}{\sum }}\overset{i_{3}+\mu -1}{\underset{i_{5}=0}{\sum }}\overset{i_{2}}{\underset{i_{6}=0}{\sum }}\frac{\mu^{i_{1}+i_{2}+2i_{3}+\mu }\kappa^{i_{1}+i_{3}}}{i_{1}!i_{2}!i_{3}!\Gamma \left(i_{3}+\mu \right)}{\left(\frac{1+\kappa }{w}\right)}^{i_{2}+i_{3}+\mu }\left(\begin{array}{c} i_{2}\\ i_{6} \end{array}\right)\left(\begin{array}{c} i_{3}+\mu -1\\ i_{5} \end{array}\right)\overset{+\infty }{\underset{0}{\int }}{\left(\frac{t^{2}}{\beta }\right)}^{\frac{i_{2}+2i_{3}+i_{6}-i_{5}+2\mu -1}{2}}\cdot \;\left.\cdot {\left(\frac{t^{2}}{\beta }+c\right)}^{\frac{i_{2}+i_{5}-i_{6}+1}{2}}e^{-\;\frac{t^{2}}{2}-2\frac{\mu \left(1+\kappa \right)}{w}\left(\frac{t^{2}}{\beta }\right)}K_{i_{5}+i_{6}-i_{2}+1}\left(\frac{2\mu \left(1+\kappa \right)}{w}\sqrt{\frac{t^{2}}{\beta }\left(\frac{t^{2}}{\beta }+c\right)}\right)dt\right].$$

Expression (22) contains the integrals *J*_1_, *J*_2_, and *J*_3_.

(23) $$J_{1}=\overset{+\infty }{\underset{0}{\int }}e^{-\frac{t^{2}}{2}}dt,\;\;J_{2}=\overset{+\infty }{\underset{0}{\int }}{\left(\frac{t^{2}}{\beta }\right)}^{i_{4}}e^{-\frac{\mu \left(1+\kappa \right)}{w}\left(\frac{t^{2}}{\beta }\right)}e^{-\frac{t^{2}}{2}}dt,\;\;J_{3}=\int_{0}^{+\infty }{\left(\frac{t^{2}}{\beta }\right)}^{\frac{i_{2}+2i_{3}+i_{6}-i_{5}+2\mu -1}{2}}\cdot \;\cdot {\left(\frac{t^{2}}{\beta }+c\right)}^{\frac{i_{2}+i_{5}-i_{6}+1}{2}}e^{-\frac{t^{2}}{2}-\frac{2\mu \left(1+\kappa \right)}{w}\left(\frac{t^{2}}{\beta }\right)}K_{i_{5}+i_{6}-i_{2}+1}\left(\frac{2\mu \left(1+\kappa \right)}{w}\sqrt{\frac{t^{2}}{\beta }\left(\frac{t^{2}}{\beta }+c\right)}\right)dt.$$

Using the formula [22], Equation (3.321.3):

(24) $$\overset{+\infty }{\underset{0}{\int }}e^{-q^{2}x^{2}}dx=\frac{\sqrt{\pi }}{2q}.$$

the integral *J*_1_ from expression (23) can be written in the following form:

(25) $$J_{1}=\frac{\sqrt{2\pi }}{2}.$$

By substituting *t*^2^/β = *s*, the integral *J*_2_ from expression (23) takes the following form:

(26) $$J_{2}=2^{i_{4}-1/2}\sqrt{\beta }{\left(\frac{w}{2\mu \left(1+\kappa \right)+\beta w}\right)}^{i_{4}+1/2}\Gamma \left(i_{4}+1/2\right).$$

Using the same substitution, integral *J*_3_ from (23) becomes:

(27) $$J_{3}=\frac{\sqrt{\beta }}{2}\int_{0}^{+\infty }s^{\frac{i_{2}+2i_{3}+i_{6}-i_{5}+2\mu -2}{2}}{\left(s+c\right)}^{\frac{i_{2}+i_{5}-i_{6}+1}{2}}K_{i_{5}+i_{6}-i_{2}+1}\left(\frac{2\mu \left(1+\kappa \right)}{w}\sqrt{s\left(s+c\right)}\right)e^{-\frac{4\mu \left(1+\kappa \right)+\beta w}{2w}s}ds.$$

By applying the properties of the second-order Bessel function *K*_v_(x) [22], Equation (8.432.6):

(28) $$K_{v}\left(z\right)=\frac{1}{2}{\left(\frac{z}{2}\right)}^{v}\int_{0}^{\infty }\frac{e^{-t-\frac{z^{2}}{4t}}}{t^{v+1}}dt.$$

the integral *J*_3_ can be expressed in the following form:

(29) $$J_{3}=\frac{\sqrt{\beta }\;\mu^{i_{5}+i_{6}-i_{2}+1}{\left(1+\kappa \right)}^{i_{5}+i_{6}-i_{2}+1}}{4w^{i_{5}+i_{6}-i_{2}+1}}\int_{0}^{\infty }t^{i_{2}-i_{5}-i_{6}-2}e^{-t}dt\int_{0}^{+\infty }s^{\frac{2i_{3}+i_{5}+3i_{6}+2\mu -i_{2}}{2}}\cdot \;\cdot {\left(c+s\right)}^{\frac{3i_{5}+i_{6}-i_{2}+3}{2}}e^{-\frac{4\mu \left(1+\kappa \right)+\beta w}{2w}s}e^{-\frac{s\left(s+c\right)}{t}{\left(\frac{\mu \left(1+\kappa \right)}{w}\right)}^{2}}ds.$$

By applying expression (16) for the binomial expansion and [22], Equation (1.211.1):

(30) $$e^{x}=\sum_{n=0}^{\infty }x^{n}/n!.$$

and, upon further evaluation, the integral *J*_3_ becomes:

(31) $$J_{3}=\sqrt{\beta }\sum_{i_{7}=0}^{\infty }\overset{\left(3i_{5}+i_{6}+2i_{7}-i_{2}+3\right)/2}{\underset{i_{8}=0}{\sum }}\frac{{\left(-1\right)}^{i_{7}}c^{\frac{3i_{5}+i_{6}+2i_{7}-2i_{8}-i_{2}+3}{2}}{\left(\mu \left(1+\kappa \right)\right)}^{i_{5}+i_{6}+2i_{7}-i_{2}+1}}{i_{7}!{\left(4\mu \left(1+\kappa \right)+\beta w\right)}^{\frac{2i_{3}+i_{5}+3i_{6}+2i_{7}+2i_{8}+2\mu -i_{2}+2}{2}}}\cdot \;\cdot 2^{\frac{2i_{3}+i_{5}+3i_{6}+2i_{7}+2i_{8}+2\mu -i_{2}-2}{2}}w^{\frac{i_{2}+2i_{3}+i_{6}+2i_{8}-i_{5}-2i_{7}+2\mu }{2}}\left(\begin{array}{c} \left(3i_{5}+i_{6}+2i_{7}-i_{2}+3\right)/2\\ i_{8} \end{array}\right)\cdot \;\cdot \Gamma \left(i_{2}-i_{5}-i_{6}-i_{7}-1\right)\Gamma \left(\frac{2i_{3}+i_{5}+3i_{6}+2i_{7}+2i_{8}-i_{2}+2\mu +2}{2}\right).$$

By substituting expressions (25) and (26) into (22), *P*_e_ becomes:

(32) $$P_{e}=\frac{1}{\sqrt{2\pi }}\left[\frac{\sqrt{2\pi }}{2}\right.-\sqrt{\beta }\;e^{-\mu \kappa }\overset{+\infty }{\underset{i_{3}=0}{\sum }}\frac{{\left(\mu \kappa \right)}^{i_{3}}}{i_{3}!}\left(\sqrt{w}\overset{i_{3}+\mu -1}{\underset{i_{4}=0}{\sum }}\frac{2^{i_{4}-1/2}{\left(\mu \left(1+\kappa \right)\right)}^{i_{4}}\Gamma \left(i_{4}+1/2\right)}{i_{4}!{\left(2\mu \left(1+\kappa \right)+\beta w\right)}^{i_{4}+1/2}}\right.+\;e^{-\mu \kappa }\cdot \;\cdot \overset{+\infty }{\underset{i_{1}=0}{\sum }}\frac{{\left(\mu \kappa \right)}^{i_{1}}}{i_{1}!}\left(\sqrt{w}\overset{i_{3}+\mu -1}{\underset{i_{4}=0}{\sum }}\frac{2^{i_{4}-1/2}{\left(\mu \left(1+\kappa \right)\right)}^{i_{4}}\Gamma \left(i_{4}+1/2\right)}{i_{4}!{\left(2\mu \left(1+\kappa \right)+\beta w\right)}^{i_{4}+1/2}}\right.-2\;\overset{i_{1}+\mu -1}{\underset{i_{2}=0}{\sum }}\overset{i_{3}+\mu -1}{\underset{i_{5}=0}{\sum }}\overset{i_{2}}{\underset{i_{6}=0}{\sum }}\overset{\infty }{\underset{i_{7}=0}{\sum }}\overset{\left(3i_{5}+i_{6}+2i_{7}-i_{2}+3\right)/2}{\underset{i_{8}=0}{\sum }}\;\cdot \frac{{\left(-1\right)}^{i_{7}}w^{\frac{i_{6}+2i_{8}-i_{2}-i_{5}-2i_{7}}{2}}{\left(\mu \left(1+\kappa \right)\right)}^{i_{3}+i_{5}+i_{6}+2i_{7}+\mu +1}c^{\frac{3i_{5}+i_{6}+2i_{7}-2i_{8}-i_{2}+3}{2}}\Gamma \left(i_{2}-i_{5}-i_{6}-i_{7}-1\right)}{2^{\frac{i_{2}-2i_{3}-i_{5}-3i_{6}-2i_{7}-2i_{8}-2\mu +2}{2}}\Gamma \left(i_{3}+\mu \right)i_{2}!i_{7}!{\left(4\mu \left(1+\kappa \right)+\beta w\right)}^{\frac{2i_{3}+i_{5}+3i_{6}+2i_{7}+2i_{8}+2\mu -i_{2}+2}{2}}}\left(\begin{array}{c} i_{2}\\ i_{6} \end{array}\right)\cdot \;\cdot \left(\begin{array}{c} i_{3}+\mu -1\\ i_{5} \end{array}\right)\Gamma \left(\frac{2i_{3}+i_{5}+3i_{6}+2i_{7}+2i_{8}-i_{2}+2\mu +2}{2}\right)\left.\left.\left.\left(\begin{array}{c} \left(3i_{5}+i_{6}+2i_{7}-i_{2}+3\right)/2\\ i_{8} \end{array}\right)\right)\right)\right].$$

For parameter values β = 2*^n^*, the considered modulation schemes include QPSK (β = 1) and BPSK (β = 2). These settings allow for the analysis of the system performance for the most commonly used digital modulation schemes in wireless communication.

### 2.3. System Performance for QPSK Modulation Using the AF Technique

**Figure 3:**
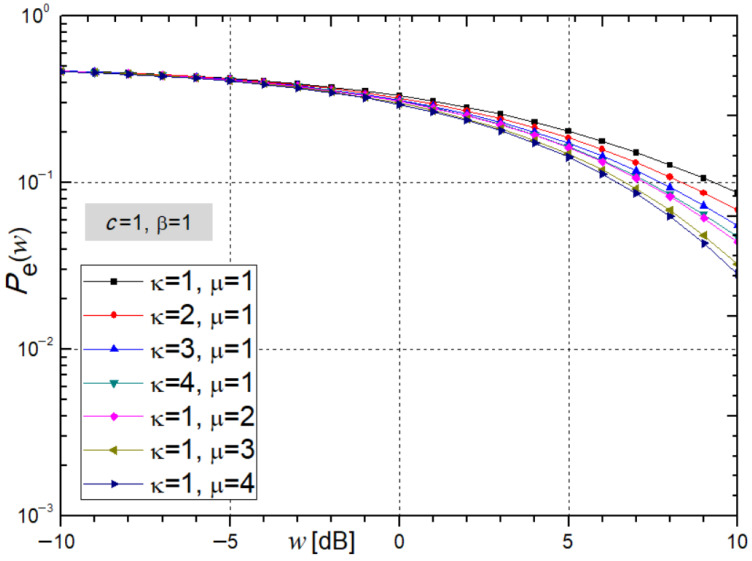
Average bit error probability from expression (32) under varying ĸ and µ, β = 1.

For quaternary QPSK modulation [24,25], when the parameter takes the value β = 2*^n^* = 1, Figure 3 illustrates the average bit error probability, obtained from expression (32), as a function of the average SNR (*w*), under varying values of the parameters ĸ and µ.

From Figure 3, it can be observed that an increase in the values of the parameters ĸ and µ leads to a decrease in the average bit error probability, indicating improved performance and increased system stability. Specifically, for ĸ = µ = 2 and an average SNR of *w* = 5 dB, the obtained *P*_e_ values calculated using *Mathematica* are 0.1912 and 0.1681.

The following Table 2 presents the required number of summation terms that need to be accumulated in order to achieve the convergence of expression (32) up to the fifth decimal place, under varying values of the system parameters ĸ and µ. This provides an analytical insight into the computational effort necessary to ensure numerical accuracy when evaluating the average bit error probability.

As the parameters ĸ and µ increase, a larger number of terms must be summed in each series to achieve convergence, resulting in a slower series convergence. For instance, when the parameter ĸ increases from 1 to 2 at an average SNR of *w* = 0 dB, the number of summation terms in the expression rises from 7^3^ = 343 to 9^3^ = 729.

### 2.4. System Performance for BPSK Modulation Using the AF Technique

**Figure 4:**
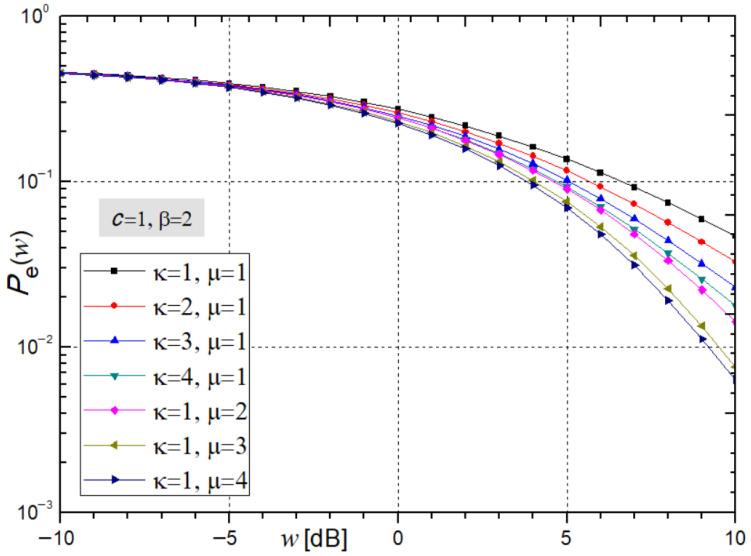
Average bit error probability from expression (32) under varying ĸ and µ, β = 2.

Figure 4 shows the average bit error probability *P*_e_ versus the average SNR (*w*) from expression (32) for varying ĸ and µ.

From the figure, it can be observed that, as the parameters ĸ and µ increase, the average bit error probability *P*_e_ at the receiver, calculated from expression (32) decreases, indicating improved system stability. For ĸ = µ = 2 and an average SNR of *w* = 5 dB, the values of *P*_e_ from expression (32) obtained using *Mathematica* are 0.1235 and 0.0974.

In addition to the graphical representation, Table 3 presents the convergence of expression (32), showing the number of series terms required to achieve an accuracy up to the fifth decimal place for varying values of the system parameters ĸ and µ.

From Table 3, it can be observed that, as the values of the parameters ĸ and µ increase, a greater number of terms in expression (32) are required to achieve the desired accuracy, resulting in a slower series convergence. This highlights the increased computational effort needed to accurately evaluate the average bit error probability for higher parameter values, reflecting the impact of channel conditions on system performance.

## 3. System Outage Probability and Average Bit Error Probability for the DF Technique

The relay transmission model of the information signal in a wireless system, where both links (S–R and R–D) are subject to κ-µ fading, is illustrated in Figure 1. The source-to-relay (S–R) link experiences κ_1_-µ_1_ fading with average power γ_1_, while the relay-to-destination (R–D) link is dominated by κ_2_-µ_2_ fading with average power γ_2_. In this section, the application of the DF protocol is considered, where the relay first decodes the signal received from the source, then re-encodes it, and subsequently forwards it to the destination [24]. This approach is also referred to in the literature as a regenerative relay. However, under unfavorable channel conditions (e.g., low SNR), the decoding process at the relay may require additional time, negatively affecting the overall temporal efficiency of the system and degrading its performance [25]. Furthermore, in the DF technique, the relay’s transmitted power depends on the so-called relay power constant *c*. For values of *c* > 1, the relay output power may be reduced, further impacting the quality of the transmission to the destination. In this chapter, based on the model in Figure 1, the system’s analytical and numerical characteristics are presented in terms of first-order statistical metrics: outage probability (*P*_out_) and average bit error probability (*P*_e_). Calculations were performed using Wolfram *Mathematica*, assuming identical parameters for both links: γ_1_ = γ_2_ = γ, μ_1_ = μ_2_ = μ, and κ_1_ = κ_2_ = κ.

### 3.1. System Outage Probability at the Receiver for the DF Technique

The system outage probability for the relay-to-destination link [26], at D, is:

(33) $$P_{out}=F_{\gamma_{eq}}\left(\gamma_{th}\right)=\overset{+\infty }{\underset{0}{\int }}P_{\mu }\left(\gamma_{1}\langle \frac{\gamma_{th}c}{\gamma_{1-}\gamma_{th}}|\gamma_{2}\right)p_{\gamma_{1}}\left(\gamma_{1}\right)d\gamma_{2}=\overset{+\infty }{\underset{0}{\int }}F_{\kappa -\mu }\left(\frac{\gamma_{th}\left(c+\gamma_{2}\right)}{\gamma_{2}}\right)p_{\kappa -\mu }\left(\gamma_{2}\right)d\gamma_{2}.$$

where *c* is a binary indicator that shows whether the relay has successfully decoded the received signal (*c* = 1) or not (*c* = 0).

Using expression (1), the PDF of the SNR under κ-µ fading is obtained as:

(34) $$p_{\kappa -\mu }\left(\gamma_{2}\right)=\frac{1}{e^{\mu k}}\overset{+\infty }{\underset{i_{3}=0}{\sum }}\frac{\mu^{2i_{3}+\mu }\kappa^{i_{3}}{\gamma_{2}}^{i_{3}+\mu -1}}{\Gamma \left(i_{3}+\mu \right)i_{3}!}{\left(\frac{1+\kappa }{w}\right)}^{i_{3}+\mu }e^{-\frac{\mu \left(1+k\right)}{w}\gamma_{2}}.$$

Applying expression (6) yields the SNR CDF at D under κ-µ fading:

(35) $$F_{\kappa -\mu }\left(\frac{\gamma_{th}\left(c+\gamma_{2}\right)}{\gamma_{2}}\right)=\frac{1}{e^{\mu \kappa }}\overset{+\infty }{\underset{i_{1}=0}{\sum }}\frac{{\left(\mu \kappa \right)}^{i_{1}}}{i_{1}!}(1-e^{-\frac{\mu \gamma_{th}\left(1+\kappa \right)\left(c+\gamma_{2}\right)}{w\gamma_{2}}}\overset{i_{1}+\mu -1}{\underset{i_{2}=0}{\sum }}\frac{1}{i_{2}!}{\left(\frac{\mu \gamma_{th}\left(1+\kappa \right)\left(c+\gamma_{2}\right)}{w\gamma_{2}}\right)}^{i_{2}}).$$

By applying expressions (34), (35), and (16) to (33), the system outage probability at the receiver for signal transmission with κ-µ fading on both links is obtained as:

(36) $$P_{out}=\frac{1}{e^{2\mu \kappa }}\overset{+\infty }{\underset{i_{1}=0}{\sum }}\overset{+\infty }{\underset{i_{3}=0}{\sum }}\frac{\mu^{i_{1}+2i_{3}+\mu }\kappa^{i_{1}+i_{3}}}{\Gamma \left(i_{3}+\mu \right)i_{1}!i_{3}!}{\left(\frac{1+\kappa }{w}\right)}^{i_{3}+\mu }\left(\overset{+\infty }{\underset{0}{\int }}{\gamma_{2}}^{i_{3}+\mu -1}e^{-\frac{\mu \left(1+\kappa \right)}{w}\gamma_{2}}d\gamma_{2}\right.-e^{-\frac{\mu \left(1+\kappa \right)\gamma_{th}}{w}}\cdot \;\cdot \overset{i_{1}+\mu -1}{\underset{i_{2}=0}{\sum }}\overset{i_{2}}{\underset{i_{4}=0}{\sum }}\left(\begin{array}{c} i_{2}\\ i_{4} \end{array}\right)\left.\frac{c^{i_{2}-i_{4}}}{i_{2}!}{\left(\frac{\mu \gamma_{th}\left(1+\kappa \right)}{w}\right)}^{i_{2}}\overset{+\infty }{\underset{0}{\int }}{\gamma_{2}}^{i_{3}+i_{4}-i_{2}+\mu -1}e^{-\frac{\mu \left(1+\kappa \right)}{w}\gamma_{2}-\frac{\mu c\left(1+\kappa \right)\gamma_{th}}{w\gamma_{2}}}d\gamma_{2}\right).$$

In expression (36), there are integrals *I*_1_ and *I*_2_. By applying suitable substitutions and further simplification, the integral *I*_1_ from (36) can be expressed as:

(37) $$I_{1}=\overset{+\infty }{\underset{0}{\int }}{\gamma_{2}}^{i_{3}+\mu -1}e^{-\frac{\mu \left(1+\kappa \right)}{w}\gamma_{2}}d\gamma_{2}={\left(\frac{w}{\mu \left(1+\kappa \right)}\right)}^{i_{3}+\mu }\Gamma \left(i_{3}+\mu \right).$$

Using expression (18), the integral *I*_2_ in (36) can be written as:

(38) $$I_{2}=\overset{+\infty }{\underset{0}{\int }}{\gamma_{2}}^{i_{3}+i_{4}-i_{2}+\mu -1}e^{-\frac{\mu \left(1+\kappa \right)}{w}\gamma_{2}-\frac{\mu c\left(1+\kappa \right)\gamma_{th}}{w\gamma_{2}}}d\gamma_{2}=2{\left(c\gamma_{th}\right)}^{\frac{i_{3}+i_{4}-i_{2}+\mu }{2}}K_{i_{3}+i_{4}-i_{2}+\mu }\left(\frac{2\mu \left(1+\kappa \right)\sqrt{c}}{w}\sqrt{\gamma_{th}}\right).$$

By substituting expressions (37) and (38) into (36), the final form of the system outage probability at receiver D for κ-µ fading on both links is obtained:

(39) $$P_{out}=\frac{1}{e^{2\mu \kappa }}\overset{+\infty }{\underset{i_{1}=0}{\sum }}\overset{+\infty }{\underset{i_{3}=0}{\sum }}\frac{\mu^{i_{1}+2i_{3}+\mu }\kappa^{i_{1}+i_{3}}}{\Gamma \left(i_{3}+\mu \right)i_{1}!i_{3}!}{\left(\frac{1+\kappa }{w}\right)}^{i_{3}+\mu }\left({\left(\frac{w}{\mu \left(1+\kappa \right)}\right)}^{i_{3}+\mu }\Gamma \left(i_{3}+\mu \right)\right.-2e^{-\frac{\mu \left(1+\kappa \right)\gamma_{th}}{w}}\cdot \;\overset{i_{1}+\mu -1}{\underset{i_{2}=0}{\sum }}\overset{i_{2}}{\underset{i_{4}=0}{\sum }}\left(\begin{array}{c} i_{2}\\ i_{4} \end{array}\right)\left.\frac{1}{i_{2}!}{\left(\frac{\mu \left(1+\kappa \right)}{w}\right)}^{i_{2}}c^{\frac{i_{2}+i_{3}-i_{4}+\mu }{2}}{\gamma_{th}}^{\frac{i_{2}+i_{3}+i_{4}+\mu }{2}}K_{i_{3}+i_{4}-i_{2}+\mu }\left(\frac{2\mu \left(1+\kappa \right)\sqrt{c}}{w}\sqrt{\gamma_{th}}\right)\right).$$

The computational complexity of the outage probability expression (39) is determined by the structure of its nested summations. The expression consists of an outer double summation over the indices *i*_1_ and *i*_3_, and an inner double summation over the indices *i*_2_ and *i*_4_, where the upper bound of i_2_ depends on i_1_ and the upper bound of *i*_4_ depends on i_2_. For numerical evaluation, the infinite summations are truncated to a finite order N. As a result, the outer summations over *i*_1_ and *i*_3_ require O(N^2^) operations, while, for each outer summation term, the inner summations over *i*_2_ and i_4_ also require O(N^2^) operations. Since all remaining computations, including Gamma functions, exponential terms, power operations, and modified Bessel functions, are evaluated in constant time per summation term under fixed-precision arithmetic, the overall worst-case computational complexity of the outage probability expression is O(N^4^). This follows directly from the Big-O rule that multiplying two quadratic complexities results in a fourth-order complexity. Although the worst-case computational complexity of the outage probability expression is O(N^4^) due to the four nested summations, this bound is pessimistic and does not reflect practical evaluation. The inner summations converge rapidly because higher-order terms decay quickly as a result of factorial terms in the denominators, exponential attenuation, and the asymptotic decay of the modified Bessel function. Consequently, for sufficiently large outer indices, only a small number of inner summation terms contribute meaningfully to the final result, allowing the early truncation of the innermost loop. As a result, the effective range of the inner summations becomes bounded by a constant or grows much more slowly than N. This reduces the average computational cost of the inner summations to O(N), while the outer double summation still requires O(N^2^) operations. Therefore, by exploiting convergence and adaptive truncation, the effective computational complexity of the outage probability evaluation is reduced to approximately O(N^3^) in practice. On the other side, the space complexity of the given outage probability expression is low compared to its time complexity, considering that computation is performed by evaluating nested summations in a sequential manner, and, at any point, only a constant number of scalar variables (such as summation indices, intermediate products, and function outputs) need to be stored. Therefore, in the case when summation terms are computed on the fly without storing all intermediate results, the space complexity is O(1).

**Figure 5:**
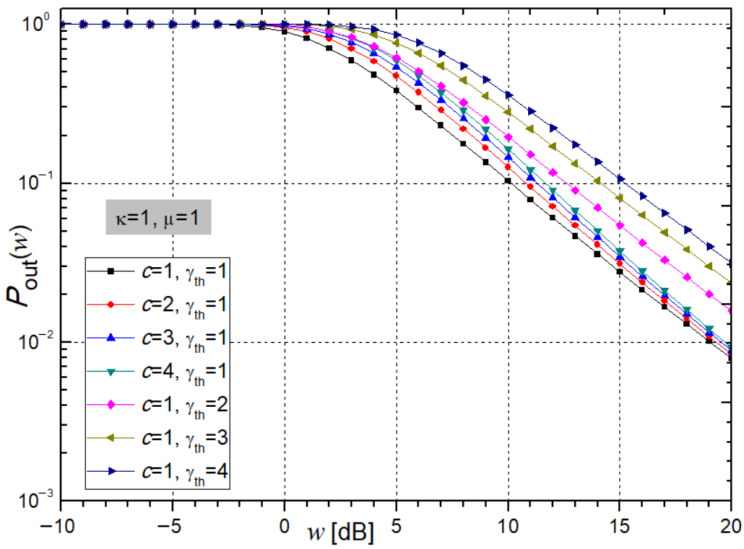
System outage probability from expression (39) under varying parameters c and γ_th_.

**Figure 6:**
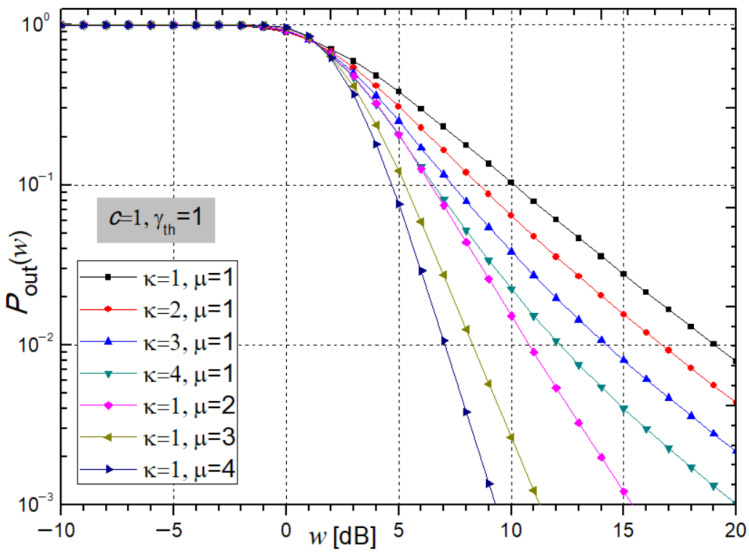
System outage probability from expression (39) under varying parameters ĸ and µ.

Figure 5 and Figure 6 illustrate the system’s *P*_out_ at the receiver, calculated from expression (39), as a function of the average SNR (*w*).

Figure 5 illustrates the variation in the system outage probability *P*_out_ as a function of the SNR during relay-based signal transmission, obtained from expression (39), under increasing values of the parameters *c* and γ_th_. As the relay power constant *c* increases, *P*_out_ rises more gradually, while the system becomes less stable. Similarly, an increase in γ_th_ leads to a higher *P*_out_, further degrading system stability. For *c* = 2 and γ_th_ = 2 at an average SNR of *w* = 5 dB, the values of *P*_out_, obtained using *Mathematica*, are 0.4719 and 0.6161.

Figure 6 shows the system outage probability *P*_out_ from expression (39) for varying µ and ĸ. As µ and ĸ increase, *P*_out_ decreases for the positive SNR, indicating improved stability. For µ = ĸ = 2 and *w* = 5 dB, *P*_out_, obtained using *Mathematica*, is 0.2053 and 0.3088.

Table 4 and Table 5 detail the number of terms in the series representation of expression (39) that need to be summed to achieve convergence with the desired accuracy, rounded to the fifth decimal place. These tables provide insight into the computational effort required for different parameter settings.

Table 4 shows the number of terms required from expression (39) for different values of *c* and γ_th_. For *w* = −10 dB and *w* = 0 dB, convergence is achieved with up to nine terms, while, at *w* = 10 dB, six terms for *c* and eight terms for γ_th_ are needed, reflecting the impact of these parameters on series convergence.

Table 5 shows the number of terms required for the convergence of expression (39) under varying parameters ĸ and µ. As ĸ and µ increase, more terms are needed in each series to achieve convergence, resulting in a slower series convergence.

### 3.2. Average Bit Error Probability for the DF Technique

By applying expression (39) into expression (21), with γ_th_ = t^2^/β, the average bit error probability at the receiver D can be expressed as follows:

(40) $$P_{e}=\frac{e^{-2\mu \kappa }}{\sqrt{2\pi }\;}\overset{+\infty }{\underset{i_{1}=0}{\sum }}\overset{+\infty }{\underset{i_{3}=0}{\sum }}\frac{\mu^{i_{1}+2i_{3}+\mu }\kappa^{i_{1}+i_{3}}}{\Gamma \left(i_{3}+\mu \right)i_{1}!i_{3}!}{\left(\frac{1+\kappa }{w}\right)}^{i_{3}+\mu }\left[{\left(\frac{w}{\mu \left(1+\kappa \right)}\right)}^{i_{3}+\mu }\Gamma \left(i_{3}+\mu \right)\overset{+\infty }{\underset{0}{\int }}\right.e^{-\frac{t^{2}}{2}}dt-2\overset{i_{1}+\mu -1}{\underset{i_{2}=0}{\sum }}\overset{i_{2}}{\underset{i_{4}=0}{\sum }}\;\left(\begin{array}{c} i_{2}\\ i_{4} \end{array}\right)\left.{\left(\frac{\mu \left(1+\kappa \right)}{w}\right)}^{i_{2}}\frac{c^{\frac{i_{2}+i_{3}-i_{4}+\mu }{2}}}{i_{2}!}\overset{+\infty }{\underset{0}{\int }}{\left(\frac{t^{2}}{\beta }\right)}^{\frac{i_{2}+i_{3}+i_{4}+\mu }{2}}e^{-\frac{\mu \left(1+\kappa \right)}{w}\frac{t^{2}}{\beta }-\frac{t^{2}}{2}}K_{i_{3}+i_{4}-i_{2}+\mu }\left(\frac{2\mu \left(1+\kappa \right)\sqrt{c}}{w}\sqrt{\frac{t^{2}}{\beta }}\right)dt\right].$$

By applying the substitution t^2^/β = s^2^, the integral *J*_2_ in expression (40) can be written in the following form:

(41) $$J_{2}=\overset{+\infty }{\underset{0}{\int }}{\left(\frac{t^{2}}{\beta }\right)}^{\frac{i_{2}+i_{3}+i_{4}+\mu }{2}}e^{-\frac{\mu \left(1+\kappa \right)}{w}\frac{t^{2}}{\beta }-\frac{t^{2}}{2}}K_{i_{3}+i_{4}-i_{2}+\mu }\left(\frac{2\mu \left(1+\kappa \right)\sqrt{c}}{w}\sqrt{\frac{t^{2}}{\beta }}\right)dt=\;=\sqrt{\beta }\overset{+\infty }{\underset{0}{\int }}s^{i_{2}+i_{3}+i_{4}+\mu }e^{-\frac{2\mu \left(1+\kappa \right)+\beta w}{2w}s^{2}}K_{i_{3}+i_{4}-i_{2}+\mu }\left(\frac{2\mu \left(1+\kappa \right)\sqrt{c}}{w}\;s\right)ds.$$

By applying the formula [22], Equation (6.631.3):

(42) $$\overset{+\infty }{\underset{0}{\int }}x^{\mu }e^{-\alpha x^{2}}K_{v}\left(\beta x\right)dx=\frac{1}{2}\alpha^{-\frac{1}{2}\mu }\beta^{-1}\Gamma \left(\left(1+v+\mu \right)/2\right)\Gamma \left(\left(1-v+\mu \right)/2\right)e^{\frac{\beta^{2}}{8\alpha }}W_{-\frac{1}{2}\mu ,\frac{1}{2}v}\left(\frac{\beta^{2}}{4\alpha }\right).$$

to expression (41), where *W*_κ,μ_(z) is the Whittaker function, the integral *J*_2_ becomes:

(43) $$J_{2}=\sqrt{\frac{\beta }{c}}\frac{\;2^{\left(i_{2}+i_{3}+i_{4}+\mu -4\right)/2}w^{\left(i_{2}+i_{3}+i_{4}+\mu +2\right)/2}\Gamma \left(i_{3}+i_{4}+\mu +1/2\right)}{\mu \left(1+\kappa \right){\left(2\mu \left(1+\kappa \right)+\beta w\right)}^{\left(i_{2}+i_{3}+i_{4}+\mu \right)/2}}\cdot \;\cdot \Gamma \left(i_{2}+1/2\right)e^{\frac{\mu^{2}{\left(1+\kappa \right)}^{2}c}{w\left(2\mu \left(1+\kappa \right)+\beta w\right)}}W_{-\frac{i_{2}+i_{3}+i_{4}+\mu }{2},\frac{i_{3}+i_{4}-i_{2}+\mu }{2}}\left(\frac{2\mu^{2}{\left(1+\kappa \right)}^{2}c}{w\left(2\mu \left(1+\kappa \right)+\beta w\right)}\right).$$

The Whittaker function can be expressed using the confluent hypergeometric function of the first kind [27], Equation (13.1.33):

(44) $$W_{\kappa ,m}\left(z\right)=e^{-z/2}z^{m+1/2}U\left(m-\kappa +1/2,1+2m;z\right).$$

The confluent hypergeometric function of the first kind can be expressed as [28]:

(45) U=Ψα,b,z=F11α,b;z=∑i=0+∞aibizii!.

where _1_*F*_1_(α,b;z) is the Kummer confluent hypergeometric function of the first kind and *a_i_* i *b_i_* are Pochhammer symbols. Using expressions (44) and (45), the Whittaker function in expression (43) can be written in the following form:

(46) $$W_{-\frac{i_{2}+i_{3}+i_{4}+\mu }{2},\frac{i_{3}+i_{4}-i_{2}+\mu }{2}}\left(\frac{2\mu^{2}{\left(1+\kappa \right)}^{2}c}{w\left(2\mu \left(1+\kappa \right)+\beta w\right)}\right)=e^{-\frac{\mu^{2}{\left(1+\kappa \right)}^{2}c}{w\left(2\mu \left(1+\kappa \right)+\beta w\right)}}\cdot \;\cdot \sum_{i_{5}=0}^{+\infty }\frac{{\left(i_{3}+i_{4}+\mu +1/2\right)}_{i_{5}}}{i_{5}!{\left(i_{3}+i_{4}-i_{2}+\mu +1\right)}_{i_{5}}}{\left(\frac{2\mu^{2}{\left(1+\kappa \right)}^{2}c}{w\left(2\mu \left(1+\kappa \right)+\beta w\right)}\right)}^{\frac{i_{3}+i_{4}+2i_{5}-i_{2}+\mu +1}{2}}.$$

By applying expressions (46) to expression (43) and using the properties of the Pochhammer symbols $x_{n}=\Gamma \left(x+n\right)/\Gamma \left(x\right)$, the integral *J*_2_ can be written as:

(47) $$J_{2}=\sqrt{\beta }\sum_{i_{5}=0}^{+\infty }\frac{2^{i_{3}+i_{4}+i_{5}+\mu -3/2}{\left(\mu \left(1+\kappa \right)\sqrt{c}\right)}^{i_{3}+i_{4}+2i_{5}-i_{2}+\mu }\Gamma \left(i_{2}+1/2\right)\Gamma \left(i_{3}+i_{4}+i_{5}+\mu +1/2\right)}{{\left(2\mu \left(1+\kappa \right)+\beta w\right)}^{i_{3}+i_{4}+i_{5}+\mu +1/2}{\left(i_{3}+i_{4}-i_{2}+\mu +1\right)}_{i_{5}}i_{5}!w^{i_{5}-i_{2}-1/2}}.$$

By substituting expressions (25) for integral *J*_1_ from (40) and (47) for integral *J*_2_ into expression (40), we obtain the expression for the average bit error probability *P*_e_ at the receiver for signal transmission modeled by the κ-μ distribution using the DF technique:

(48) $$P_{e}=\frac{1}{2\sqrt{2\pi }\;e^{2\mu \kappa }}\overset{+\infty }{\underset{i_{1}=0}{\sum }}\overset{+\infty }{\underset{i_{3}=0}{\sum }}\frac{\mu^{i_{1}+2i_{3}+\mu }\kappa^{i_{1}+i_{3}}}{\Gamma \left(i_{3}+\mu \right)i_{1}!i_{3}!}{\left(\frac{1+\kappa }{w}\right)}^{i_{3}+\mu }\left[\sqrt{2\pi }{\left(\frac{w}{\mu \left(1+\kappa \right)}\right)}^{i_{3}+\mu }\;\right.\cdot \;\cdot \Gamma \left(i_{3}+\mu \right)-\sqrt{\beta }\overset{i_{1}+\mu -1}{\underset{i_{2}=0}{\sum }}\overset{i_{2}}{\underset{i_{4}=0}{\sum }}\sum_{i_{5}=0}^{+\infty }\left(\begin{array}{c} i_{2}\\ i_{4} \end{array}\right)\frac{{\left(\mu \left(1+\kappa \right)\right)}^{i_{3}+i_{4}+2i_{5}+\mu }c^{i_{3}+i_{5}+\mu }\Gamma \left(i_{2}+1/2\right)}{{\left(i_{3}+i_{4}-i_{2}+\mu +1\right)}_{i_{5}}}\cdot \;\cdot \frac{\Gamma \left(i_{3}+i_{4}+i_{5}+\mu +1/2\right)}{w^{i_{5}-1/2}i_{2}!i_{5}!}\left.{\left(\frac{2}{2\mu \left(1+\kappa \right)+\beta w}\right)}^{i_{3}+i_{4}+i_{5}+\mu +1/2}\right].$$

When it comes to expression (48)’s computational complexity, we use the same reasoning as for (39). While the outer double summation has the same complexity as in the previous case, O(N^2^), for the outer summation term, the inner triple summation requires O(N^3^) operations. Since all remaining components of the expression, including Gamma functions, power terms, and exponential factors, are evaluated in constant time per summation term under fixed-precision arithmetic, the overall worst-case computational complexity is given by the product of the outer and inner complexities, resulting in an O(N^5^) time complexity. In practice, however, the effective computational cost is typically much lower than this worst-case bound due to the rapid convergence of the series. The presence of factorial terms, Gamma functions in the denominator, and decaying power terms causes higher-order summation terms to contribute negligibly. By exploiting convergence and applying adaptive truncation to the inner summations, the effective complexity can often be reduced to approximately O(N^4^) or lower in practical implementations. In this case, the space complexity is also O(1) under the same assumptions as for expression (39).

For the parameter values β = 2^n^, QPSK modulation (*n* = 0, β = 1) and BPSK modulation (*n* = 1, β = 2) are considered. For these two modulation schemes, the next section presents the graphical and tabular results based on expression (48).

### 3.3. System Performance for QPSK Modulation Using the DF Technique

**Figure 7:**
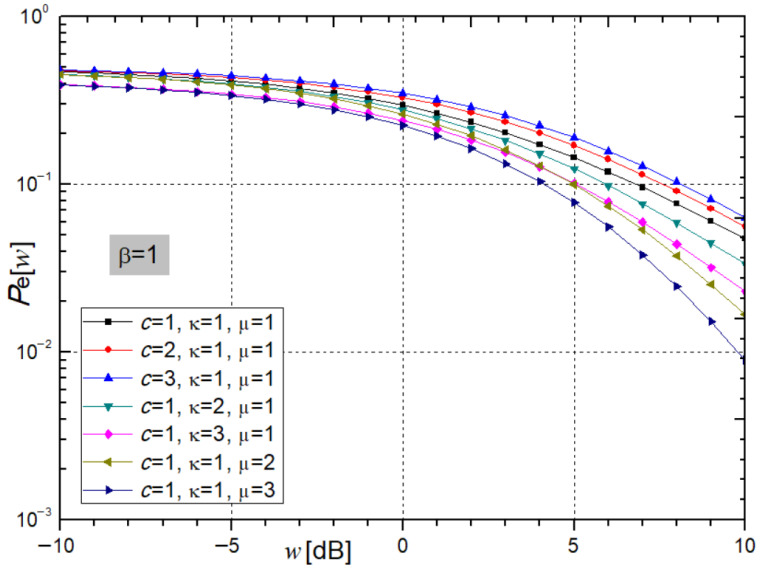
Average Bit Error Probability from expression (48) for varying parameters c, κ, and µ, β = 1.

Figure 7 presents a graphical representation of expression (48), showing the average bit error probability at the receiver as a function of the average SNR. The figure illustrates the impact of varying the κ and µ parameters on the performance of quadrature QPSK modulation (β = 1).

The average bit error probability (*P*_e_) at the receiver, derived from Expression (48), exhibits a slight increase with rising values of the relay power parameter ccc, indicating a modest degradation in system performance. Conversely, an increase in the fading parameters κ and μ results in a decrease in *P*_e_, reflecting the enhanced system performance and improved stability. For the parameter set *c* = κ = μ = 2 and an average SNR of *w* = 5 dB, the corresponding *P*_e_ values are 0.1707, 0.1237, and 0.0996.

Table 6 shows the number of series terms required for the convergence of expression (48) to achieve an accuracy rounded to the fifth decimal place.

Table 6 shows that the number of terms required for the convergence of expression (48) ranges from 6 to 13. Increasing the value of parameter *c* reduces the number of terms needed and accelerates the convergence of the expression, whereas increasing the parameters κ and μ slows down the convergence, requiring the summation of more terms to achieve the desired accuracy. These results highlight the significant impact of system parameters on the numerical efficiency of relay system performance calculations.

### 3.4. System Performance for BPSK Modulation Using the DF Technique

**Figure 8:**
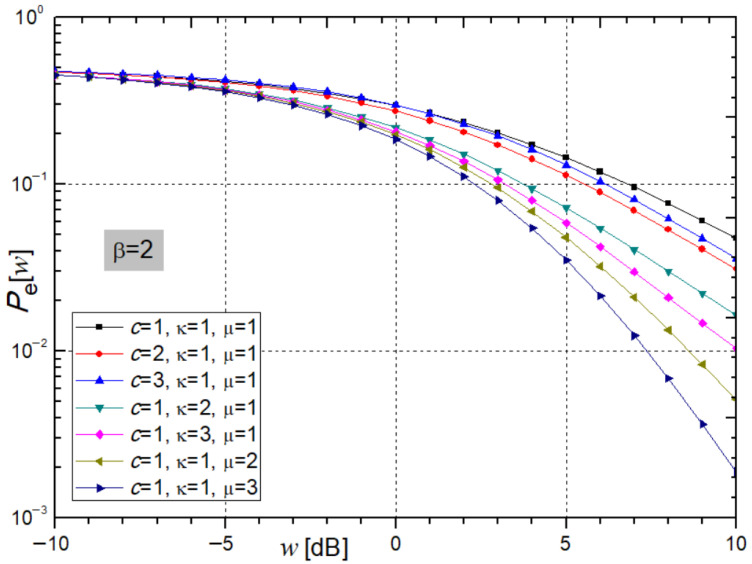
Average Bit Error Probability from expression (48) for varying parameters c, κ, and µ, β = 2.

For BPSK modulation, Figure 8 illustrates the graph of expression (48) for the average bit error probability (*P*_e_) at the receiver, as a function of the average SNR, with varying system parameters *c*, κ, and μ.

At the receiver, the average bit error probability (*P*_e_) increases more slowly with the rise in parameter *c*, indicating a degraded performance and partial system instability. In contrast, increasing the parameters κ and μ reduces *P*_e_ at the receiver, resulting in improved system stability. For the parameter values *c* = κ = μ = 2 and an average SNR of *w* = 5 dB, *P*_e_ equals 0.1133, 0.0723, and 0.0479, respectively.

In addition to the graphical representation, Table 7 presents the number of terms from expression (48) required for convergence, indicating how many terms must be summed to achieve a precision up to the fifth decimal place.

Table 7 shows the number of terms required for the convergence of expression (48) with varying *c*, κ, and µ. Higher *c* values allow convergence with fewer terms, while increasing κ and µ slows convergence, requiring more terms to achieve the desired precision.

## 4. Numerical Analysis and Comparison of Results Obtained Using AF and DF Techniques for Signal Transmission Under κ-µ Fading

In order to evaluate and improve the system performance, this chapter presents a detailed numerical analysis and comparative study of key first-order statistical metrics: system outage probability (*P*_out_) and average bit error probability (*P*_e_). The comparison is based on signal transmission models from the transmitter to the receiver in a κ-µ fading environment, without the application of diversity techniques. The analysis includes a percentage-based comparison of the results from Chapters 2 and 3, using standard criteria for assessing *P*_out_ and *P*_e_, with the application of AF and DF relay techniques. The channel parameters considered are set as *c* = κ = µ = γ_th_ = 2, with an average SNR value of *w* = 5 dB, and the numerical calculations are performed using Mathematica [19]. The meaning of the selected channel parameter values used for performance comparison is as follows:

‑ *c* = 2: Relay power constant (*c* > 1, reduced retransmit power);
‑ ĸ = 2: Dominant component magnitude;
‑ µ = 2: Higher fading severity (larger number of scattering clusters);
‑ γ_th_ = 2: Lower receiver sensitivity (for example, more noise present in the receiver front-end).

For this case, using AF and DF transmission based on the model in Figure 1, the comparison results from Section 2 and Section 3 are presented in Table 8.

The analysis and comparison of the results presented in Table 8 reveal a clear advantage of employing the DF technique over the AF approach. Specifically, the system outage probability (*P*_out_) is significantly lower when the DF technique is used, indicating the higher reliability and stability of signal transmission. Furthermore, based on the comparison of the average bit error probability (*P*_e_) values for quadrature QPSK and binary BPSK modulation, it is evident that the DF technique provides better error resilience and more accurate data transmission. The system modeled in Figure 1 exhibits lower *P*_e_ values with the application of the DF technique compared to the AF method, demonstrating improved performance in terms of reception quality and transmission stability. From the above, it can be concluded that the DF approach is superior to AF, as it allows for signal regeneration at the relay, reduces noise propagation, and leads to a significant enhancement in key system parameters, including the reliability, stability, and overall performance of the wireless communication channel.

## 5. GenAI-Empowered Framework for Fading-Aware Network Maintenance

### 5.1. Related Works and Motivation

The outcome presented in this paper builds upon a coherent line of our previous research focused on integrating GenAI with automated network design, experimentation, management, and security. In [29], we introduced the synergy of model-driven engineering (MDE) and LLMs, where model-based representations serve as compact experiment configurations, verifiable intermediate artifacts, and human-interpretable interfaces, implemented using Eclipse Modeling Framework (EMF) and Object Constraint Language (OCL). Such an approach was used for performance-aware network planning in the design phase, considering the impact of fading and co-channel interference. This foundation was extended in [30] by incorporating RAG to efficiently process large textual inputs such as network standards and reference architectures to be taken into account as constraints. A complementary metamodeling approach for the automated abstraction of network experiments from textual descriptions was presented in [31], while [32] demonstrated the use of VLMs to extract actionable knowledge from performance and topology diagrams. This way, we enabled the extraction of the key aspects and implicit topology based on network-related requirements specifications. On the other side, works from [33,34] explored agent-based AI approaches for automated network infrastructure and IoT system management in run-time, focusing on resilience and sustainability. Together, these works establish the methodological and technological basis for the contributions of this paper.

On the other hand, it can be also noticed that the recent research by other authors and research groups also exhibits a growing adoption of GenAI for advanced network management through diverse methodological perspectives. Agentic and multi-agent LLM frameworks enable intent-driven, adaptive, and accessible network control, supporting autonomous orchestration and dynamic decision-making in complex telecom environments [35,36]. Complementary LLM-based approaches address analytics and reasoning over heterogeneous telecom and knowledge-graph data to enhance operational intelligence [37]. Vision–language models have been explored both for the visual understanding of telecom artifacts, such as image-to-UML conversion, and for autonomous operator-style agents handling multimodal tasks in demanding domains [38,39]. In parallel, retrieval-augmented generation has been applied to improve the interpretation of telecom standards and specifications, ensuring compliant and explainable automation [40]. Together, these studies show strong progress toward intelligent network automation while motivating unified, end-to-end, and security-aware solutions spanning both design-time and run-time management.

The purpose of our GenAI adoption in this paper is not to derive closed-form expressions, but rather as a supporting tool which aims to reduce the cognitive overhead of network-management-related operations resulting from the complexity of the underlying infrastructure, leading to the significant time and resources needed. Additionally, we also aim to bridge the gap between design-time and run-time network management, enabling the flexible and continuous evolution of network deployments. By unifying model-driven design artifacts with agentic GenAI-based reasoning at run time, the proposed approach supports seamless transitions from planning and experimentation to deployment, monitoring, and adaptation. Such integration allows network configurations, performance insights, and operational feedback to co-evolve throughout the network lifecycle, facilitating iterative refinement and informed decision-making. As a result, the framework overcomes the limitations of static, phase-specific solutions and enables more adaptive, scalable, and evolution-aware GenAI-assisted network management. In the presented case study, the proposed solution mainly targets the outage probability and average bit error probability reduction by selecting the suitable actions from the list of possible ones, as well as parametrizing and generating corresponding commands to execute those actions against the target infrastructure. The decisions for these actions are based on the estimated performance values, based on the calculation with respect to the derived closed form outage probability expression.

### 5.2. Workflow Overview

**Figure 9:**
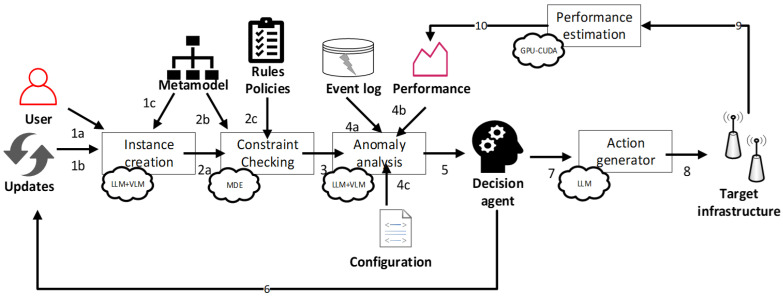
GenAI-based run-time network maintenance workflow: (1a) user-driven updates and intents; (1b) system-triggered updates due to network events and topology changes; (1c/2b) Ecore metamodel as structural input defining covered topology and environment aspects; (2a) metamodel-driven instance creation/alignment of model based on updates; (2c) OCL rules defining network policies and operational constraints; (3) outcome of policy and constraint checking; (4a) historical records of event logs used for anomaly detection; (4b) run-time performance measurements; (4c) configuration and state information used for anomaly analysis; (5) consolidated results of run-time analysis and policy evaluation; (6) management and adaptation decisions; (7) translation into platform-specific control commands; (8) execution on the target infrastructure; (9) updated system in action; and (10) GPU-accelerated performance estimation supporting a closed-loop feedback mechanism for continuous run-time adaptation.

Figure 9 illustrates a GenAI-enabled workflow designed to support continuous network analysis, decision-making, and adaptive management across the operational lifecycle of wireless infrastructures.

The workflow is initiated by the continuous ingestion of updates from the network, including new requirements to be considered and updates of the existing requirements, covering the addition/removal of devices and services, as well as the configuration and parameter value changes. These inputs provide real-time visibility into the network behavior and act as triggers for the subsequent management and adaptation processes. In parallel, operators can provide high-level, freeform textual descriptions of network experiments or management intents, including assumptions related to fading conditions, interference characteristics, infrastructure parameters, and traffic demand.

Using Large Language Models (LLMs) and Vision–Language Models (VLMs), these heterogeneous inputs, ranging from textual specifications to visual artifacts such as topology diagrams or deployment schematics, are interpreted and transformed into structured network and experiment instances. These instances are generated in accordance with predefined metamodels, ensuring a consistent and formal representation of network elements, configurations, and operational scenarios. The underlying metamodel relies on work from [29], while the methodology for automated metamodel generation was described in [31]. This step enables the seamless integration between human intent, analytical models, and machine-executable representations. Each generated or updated instance is then subjected to constraint checking, where model-driven engineering techniques are used to enforce predefined rules, policies, and operational boundaries. This validation step ensures the logical consistency, completeness, and feasibility of the proposed updates and configuration changes before further processing. Once validated, the workflow proceeds with the anomaly analysis, which combines the live event log monitoring data with analytically derived performance indicators, such as outage probability and average bit error probability-related QoS metrics. These computations can be efficiently executed using GPU-accelerated engines, enabling fast evaluation even under complex channel and traffic conditions, based on our prior works from [41]. Additionally, anomalies can be detected within network configuration files which are about to be applied.

The outcomes of constraint checking and run-time performance analysis are forwarded to an LLM-based decision and planning agent. This agent synthesizes the analytical results, historical context, and current network conditions to determine whether performance objectives or QoS thresholds are violated. If corrective action is required, the agent autonomously plans a sequence of adaptation steps, such as adjusting the transmission power levels, modifying the antenna parameters, or reconfiguring the network resources, in order to restore or optimize the network performance. For instance, when the computed outage probability or average bit error probability exceeds a predefined threshold, the planning agent autonomously initiates corrective measures to restore an acceptable service quality. Typical actions include incrementally increasing the transmission power (e.g., by 2 dB) or modifying the antenna azimuth (e.g., by 10°) to improve coverage and link reliability. The selected actions are translated into reconfiguration commands that are submitted to the network via a NETCONF service endpoint.

The execution of these actions is handled by a complementary planning agent that follows a structured plan and interacts with the infrastructure through a set of predefined functions. These functions enable the agent to retrieve up-to-date signal-to-noise ratio (SNR) measurements, compute *P*_out_ and/or *P*_e_, query the current cell configuration parameters, update the transmission power and antenna settings, and, finally, apply the new configuration to the target cell. Key actions include:

(1) *retrieve_radio_quality_indicators (cell_id)*—this obtains the most recent signal quality measurements (e.g., SNR) for a given cell from the network management interface;
(2) *evaluate_service_degradation (signal_metrics, qos_threshold)*—this computes the outage probability by comparing the measured signal quality against a predefined QoS threshold;
(3) *query_current_cell_parameters (cell_id)*—this fetches the active configuration of the selected cell, including the transmission power and antenna orientation;
(4) *prepare_updated_settings (cell_id, target_power, target_azimuth)*—this defines the new transmission power levels and antenna angles required to improve performance;
(5) *apply_network_reconfiguration(cell_id, power_setting, azimuth_setting)—*this enforces the updated parameters on the infrastructure through the management interface, completing the adaptation cycle.

Based on these decisions, the current model instance is updated in order to check the consistency after applying the proposed actions. Finally, once the “to-be” model instance representation is validated, an action generation module, also supported by LLMs, translates high-level plans into concrete, executable commands and configuration updates. These actions are applied to the underlying infrastructure via standard management interfaces, closing the feedback loop. Through continuous monitoring, analysis, planning, and execution, the proposed workflow enables adaptive, iterative, and evolution-aware network management at run time, allowing deployments to dynamically respond to changing conditions while maintaining a stable and satisfactory service quality.

### 5.3. Experiments and Evaluation

The experimental evaluation demonstrates the effectiveness of the proposed GenAI-enabled workflow for adaptive run-time network management under heterogeneous operating conditions. In contrast to our previous works, which relied on commercial models such as GPT-4o and o1-mini, the current framework exclusively employs open foundation models. While Meta-LLaMA-3-70B-Instruct [42] is leveraged for the model instance, constraint, and command generation, Marco-o1 [43] is adopted for reasoning and planning. On the other side, for the handling of visual information, we make use of OpenGVLab’s InternVL2-8B-MPO [44] as the underlying VLM, building upon our work from [32], which was approved as an acceptable solution when it comes to the accuracy of the results, with a manageable model size, still suitable for local deployment. This shift highlights the feasibility of replacing proprietary solutions with openly accessible models while preserving advanced reasoning and decision-making capabilities, while enabling the deployment within the organizational boundaries, which ensures a seamless adoption without risk of leaking protected assets or sensible information to third parties. In what follows, Table 9 summarizes the experiments used for the validation of the proposed GenAI-empowered network management approach, together with an average response time based on four experiment runs. However, it is assumed that constraints are created before the experiment execution, so the time needed for their generation is not taken into account within the response time. GenAI-empowered experimental outcomes are empirically verified through human review. Additional information about the representative actions used for network management in the presented cases can be found in Appendix A.

The conducted experiments evaluate the effectiveness of the proposed adaptive decision-making framework under varying channel conditions, transmission parameters, and network operational states. Each case highlights a distinct system behavior and corresponding control action.

‑ *Case 1*: This case considers a low-SNR scenario (*w* = 3 dB) under moderate fading conditions (κ = 1, µ = 4). Despite satisfying the imposed system constraints, the observed bit error probability (*P*_e_) exceeds the predefined threshold of 0.1. Consequently, the decision agent triggers a corrective action by increasing the transmission power by 2 dB. The overall response time for detecting the performance degradation and executing the adaptation is 39 s, demonstrating the system’s capability to mitigate error performance deterioration under power-limited conditions.
‑ *Case 2*: The SNR is increased to *w* = 6 dB with slightly more severe fading (µ = 3). However, under these conditions, the measured *Pe* remains below the specified threshold, indicating a satisfactory link quality. As a result, no corrective action is required, and the system maintains its current configuration. The reduced response time of 19 s reflects the absence of reconfiguration overhead.
‑ *Case 3*: This case investigates the outage-driven adaptation at a higher SNR (*w* = 9 dB) but under more challenging fading conditions (µ = 2). Although the SNR is relatively high, the outage probability (*P*_out_) exceeds the target limit of 0.01, violating the reliability requirement. To address this, the framework selects the antenna azimuth adjustment as the optimal response, improving spatial alignment and link robustness. The selection of this corrective action is justified by the fact that the transmission power increase is limited by the given constraint, so another action is selected instead. This adaptive action has a response time of 41 s, primarily due to the additional control and coordination steps involved.
‑ *Case 4*: The SNR of *w* = 5dB combined with moderate fading (µ = 3) results in an outage probability below the specified threshold. The system therefore refrains from any corrective action, maintaining a stable operation. The relatively short response time of 23 s again reflects normal monitoring without reconfiguration.
‑ *Case 5*: On the other hand, it evaluates the system resilience under fault conditions. Although two base stations are initially operational, anomaly detection mechanisms identify abnormal behavior in one of the system logs, resulting in only one fully active module. Given the constraint that at least two base stations must remain active, the decision agent initiates the activation of an additional module to preserve service continuity. This process requires 49 s, representing the most time-consuming scenario due to fault diagnosis and module activation procedures.

Overall, the experimental results from Table 9 confirm that the proposed framework effectively distinguishes between performance degradation and fault-induced issues, selecting appropriate corrective actions while avoiding unnecessary reconfigurations. The adaptive responses ensure compliance with quality-of-service and reliability constraints under diverse operational conditions. Compared to our previous works which rely on commercial OpenAI’s models [29], the execution time is slightly longer, but still at an order of magnitude of 10 s. However, locally deployable models, as an advantage, offer more flexibility when it comes to deployment and the complete control of information flow.

The overview of key prompts within the workflow is given in Table 10. The performance evaluation is reported in the final column and reflects the percentage of successful executions obtained from 20 runs (4 times under identical experimental conditions for each of the 5 cases shown in Table 9). For the model instance generation step, an execution is considered successful if the current system instance is correctly updated to produce a valid XMI model that conforms to the specified metamodel. During constraint derivation, success requires the accurate extraction of formal constraints from the reference specification and their correct formulation as Object Constraint Language (OCL) rules associated with the same metamodel. For the performance analysis phase, a successful execution is defined by the correct interpretation of the provided performance diagrams, resulting in a consistent *P*_out_ and *P_e_* trend identification. In the anomaly analysis step, success is achieved when system logs are correctly processed to identify anomalous behavior and accurately report failed components or modules. Moreover, with respect to decision making, an execution is considered successful if the selected control action—such as transmission power increase, antenna azimuth adjustment, the activation of an additional module, or configuration update—is consistent with both the current network state represented by the model instance and the observed trends. Finally, for command generation, success requires the production of syntactically valid and executable platform-specific commands that correctly target the network elements described in the model instance and effectively implement the selected action. All experiments were conducted using a maximum token budget of 4096 tokens per prompt, with default temperature settings applied. While the time needed for processing depends on the input sizes, for illustrative purposes, we also include, in the last column, the average inference time for key prompts based on the presented experiments. This evaluation methodology ensures a consistent and reproducible assessment of the proposed model-driven and AI-assisted workflow. Locally deployable models were executed on an NVIDIA A100 GPU with 80 GB of VRAM.

Across the evaluated scenarios, the workflow successfully addresses variations in the SNR, fading parameters, outage probability thresholds, and infrastructure-related events. When the analytical evaluation indicates performance degradation, the selected models reason over the current network state and autonomously derive appropriate corrective actions, such as incremental radio parameter adjustments or the activation of additional resources, while ensuring compliance with predefined constraints. Conversely, when the performance metrics remain within acceptable limits, the models correctly infer that no intervention is required, thereby avoiding unnecessary reconfigurations.

Based on empirical estimations from [29], compared to traditional workflows which usually rely on diverse tools and manual operations by human operators to bridge the gap between them, our solution reduces the time needed for a response to the significant network changes by at least one order of magnitude. Furthermore, compared to other automated network management solutions [45], the proposed GenAI-enabled methodology introduces greater flexibility and generality [29], as modifications of the network topology and design decisions do not imply the design of new models or re-training the existing ones to make it work. LLMs and VLMs are already pre-trained on huge amounts of text (and images for VLMs), giving them a strong summarization and generalization potential, even in cases of previously unseen data. The required modifications in such a case would be providing a new metamodel and set of allowed network management or corrective actions, as well as the command templates which are used for their generating, while our approach aims to provide the correct context to generative models. On the other hand, compared to other GenAI-based solutions, our approach makes use of synergy with model-driven, formally grounded methods, introducing the aspects of verifiability and explainability which has a positive impact on adopting such solutions, as it increases human trust in GenAI solutions [29,31].

## 6. Conclusions

In this work, we analyzed the performance of a single-relay wireless transmission system under κ–µ fading without line-of-sight conditions. System reliability was evaluated in terms of outage probability (*P*_out_) and average bit error probability (*P*_e_), considering both AF and DF protocols. The results indicate that the DF protocol achieves lower outage and error probabilities compared to AF, thereby enhancing stability and reliability. Moreover, increasing the κ and µ parameters improves the transmission quality, while higher threshold values γ_th_ and relay power constant *c* may degrade the system performance. Analytical derivations were validated by extensive numerical simulations, showing close agreement. Overall, the findings highlight the advantages of DF relaying in κ-µ fading environments without direct visibility. Future research may extend this analysis to multi-relay configurations, cooperative diversity schemes, and the inclusion of advanced modulation formats, providing further insights into robust system design under generalized fading conditions.

Regarding the GenAI-empowered approach to performance-aware network management and maintenance, from run-time data ingestion and analytical performance evaluation to planning and execution, we design a prompt-driven workflow executed by locally deployable models: Meta-LLaMA-3-70B-Instruct (LLM agent), InternVL2-8B-MPO (VLM agent), and Marco-o1 (decision agent). These models interpret both structured and unstructured inputs, analyze performance-related trends (outage probability, and average bit error probability) together with network state information, and select suitable adaptation strategies, which are subsequently translated into executable configuration commands. The presented results confirm that open foundation models can effectively substitute commercial alternatives such as GPT-4o and o1-mini compared to our prior works, while still enabling the explainable, flexible, and efficient continuous adaptation of wireless network deployments. Despite the promising results, at the current stage of this work, the main limitation is the occurrence of so-called hallucination effect impacts that can lead to incorrect results, in some cases affecting the success of individual steps, while the adoption of tackling mechanisms for this purpose is considered as future work.

In future research, we will also cover security-related aspects and integrate them within the workflow. Additionally, as another possible direction, we would explore synergy with Deep Reinforcement Learning (DRL)-based resource allocation frameworks for Reconfigurable Intelligent Surface-based Intelligent Sensing and Communication (RIS-ISAC) scenarios, like the one presented in [46]. In large-scale RIS-ISAC deployments, especially those involving multiple base stations and distributed RISs, centralized learning might be too costly or inefficient. Therefore, Federated Learning (FL) could be used to decentralize the learning process while maintaining the overall efficiency of the system. Multiple local RIS-ISAC units could learn and optimize their parameters independently, sharing only model updates (not raw data) with a central server. For this purpose, integration with a GenAI-based architecture that synthesizes knowledge across all distributed agents and enables them to collaborate without revealing sensitive data would be a promising research direction. This approach could help scale the RIS-ISAC system to large, distributed networks while ensuring privacy and reducing the computational burden on a central node, as well as enable continuous adaptation in dynamic, large-scale environments. Finally, due to the high computational complexity of analytical model performance evaluation expressions, we will also evaluate GenAI-based estimations and their practical usability.

## Figures and Tables

**Table 1:** Number of terms required for convergence of expression (20) for varying κ, μ, and γ_th_.

*P*_out_ (*c* = 1)	*w* = −10 dB	*w* = 0 dB	*w* = 10 dB
ĸ = 1, µ = 1, γ_th_ = 1	5	6	7
ĸ = 2, µ = 1, γ_th_ = 1	5	11	11
ĸ = 3, µ = 1, γ_th_ = 1	5	14	14
ĸ = 1, µ = 2, γ_th_ = 1	5	7	8
ĸ = 1, µ = 3, γ_th_ = 1	5	7	8
ĸ = 1, µ = 1, γ_th_ = 2	5	6	8
ĸ = 1, µ = 1, γ_th_ = 3	5	6	7

**Table 2:** Number of terms for convergence of expression (32) with varying ĸ and µ.

*P*_e_ (β = 1)	*w* = −10 dB	*w* = 0 dB	*w* = 10 dB
κ = 1, µ = 1	6	7	7
κ = 2, µ = 1	8	9	10
κ = 3, µ = 1	10	13	13
κ = 4, µ = 1	12	15	14
κ = 1, µ = 2	8	10	10
κ = 1, µ = 3	10	12	13
κ = 1, µ = 4	13	14	15

**Table 3:** Number of terms required for convergence of expression (32) for varying ĸ and µ parameters.

*P*_e_ (β = 2)	*w* = −10 dB	*w* = 0 dB	*w* = 10 dB
κ = 1, µ = 1	5	7	7
κ = 2, µ = 1	8	10	10
κ = 3, µ = 1	10	13	12
κ = 4, µ = 1	12	14	15
κ = 1, µ = 2	8	10	11
κ = 1, µ = 3	10	13	13
κ = 1, µ = 4	13	15	15

**Table 4:** Series terms required for the convergence of expression (39) as a function of *c* and γ_th_.

*P*_out_ (ĸ = 1, µ = 1)	*w* = −10 dB	*w* = 0 dB	*w* = 10 dB
*c* = 1, γ_th_ = 1	9	8	6
*c* = 2, γ_th_ = 1	9	8	7
*c* = 3, γ_th_ = 1	9	9	6
*c* = 4, γ_th_ = 1	9	9	6
*c* = 1, γ_th_ = 2	9	9	7
*c* = 1, γ_th_ = 3	9	8	8
*c* = 1, γ_th_ = 4	9	9	7

**Table 5:** Series terms required for the convergence of expression (39) as a function of κ and μ.

*P*_out_ (*c* = 1, γ_th_ = 1)	*w* = −10 dB	*w* = 0 dB	*w* = 10 dB
ĸ = 1, µ = 1	9	8	6
ĸ = 2, µ = 1	12	11	9
ĸ = 3, µ = 1	13	13	11
ĸ = 4, µ = 1	15	16	12
ĸ = 1, µ = 2	12	10	8
ĸ = 1, µ = 3	13	14	9
ĸ = 1, µ = 4	15	16	8

**Table 6:** Number of terms for convergence of expression (47) with varying *c*, κ, and µ, β = 1.

*P*_e_ (β = 1)	*w* = −10 dB	*w* = 0 dB	*w* = 10 dB
*c* = 1, ĸ = 1, µ = 1	8	12	7
*c* = 2, ĸ = 1, µ = 1	8	7	7
*c* = 3, ĸ = 1, µ = 1	7	7	6
*c* = 1, ĸ = 2, µ = 1	11	10	9
*c* = 1, ĸ = 3, µ = 1	13	12	10
*c* = 1, ĸ = 1, µ = 2	11	11	9
*c* = 1, ĸ = 1, µ = 3	13	12	10

**Table 7:** Number of terms for convergence of expression (48) with varying *c*, κ, and µ, β = 2.

*P*_e_ (β = 2)	*w* = −10 dB	*w* = 0 dB	*w* = 10 dB
*c* = 1, ĸ = 1, µ = 1	8	7	7
*c* = 2, ĸ = 1, µ = 1	8	7	6
*c* = 3, ĸ = 1, µ = 1	8	7	6
*c* = 1, ĸ = 2, µ = 1	10	10	8
*c* = 1, ĸ = 3, µ = 1	13	12	10
*c* = 1, ĸ = 1, µ = 2	11	10	7
*c* = 1, ĸ = 1, µ = 3	13	12	8

**Table 8:** *P*_out_ and *P*_e_ results from the signal transmission model in Figure 1.

Comparison Metrics	Channel Parameters: *c* = 1, ĸ = 1, µ = 1, γ_th_ = 1	Retransmission with Reduced Power (*c* = 2)	Prominent Dominant Component (ĸ = 2)	Higher Number of Scattering Clusters (µ = 2)	Lower Receiver Sensitivity (γ_th_ = 2)	Relay Type: AF/DF, Chapter
*P*_out_ [%]	60.50 38.20	------ 47.19	54.53 30.88	46.77 20.53	87.99 61.61	AF (2) DF (3)
*P*_e_ [%], QPSK	20.37 14.46	------- 17.07	19.12 12.37	16.81 9.96	----- -----	AF (2) DF (3)
*P*_e_ [%], BPSK	13.69 9.11	------ 11.33	12.35 7.23	9.74 4.79	----- -----	AF (2) DF (3)

**Table 9:** Experiments overview for GenAI-empowered network management approach.

Case	Initial Conditions	System Constraints	Threshold	Decision Outcome	Response (s)
1	*w* = 3 dB Fading: κ = 1, μ = 4	*c* = 1, β = 2, *w* ≤ 6 dB	*P*e < 0.1	*P*_e_ exceeds threshold → transmission power increased by 2 dB	39
2	*w* = 6 dB Fading: κ = 1, μ = 3			*P*_e_ below threshold; no action required	19
3	*w* = 9 dB Fading: κ = 1, μ = 2	*c* = 1, γ_th_ = 1, *w* ≤ 10 dB	*P*_out_ < 0.01	*P*_out_ exceeds threshold → antenna azimuth adjusted	41
4	*w* = 5 dB Fading: κ = 1, μ = 3			*P*_out_ below threshold; no action required	23
5	Two base stations running, Anomaly present in log for one of them	At least two base stations must remain active	–	Anomaly detected, 1/2 modules active → Additional module to be activated	49

**Table 10:** Overview of key prompts and achieved performance.

Step	Description	Output	Results
Model Instance Generation	For a given [changes], the current system [instance model] is updated to obtain a compliant representation of the network state with respect to metamodel: [metamodel]	XMI model instance	Success: 80% Time: 10.2 s
Constraint Derivation	Formal constraints are extracted from [reference specification] and expressed as Object Constraint Language (OCL) rules associated with respect to metamodel: [metamodel]	OCL rules	Success: 85% Time: 8.8 s
Performance Analysis	For the considered scenario, the outage probability and average bit error probability are evaluated by analyzing the corresponding performance visualization results: [performance diagrams]	*P*_out_ and *P*_e_ trends	Success: 80% Time: 11.3 s
Anomaly Analysis	System logs: [logs] are analyzed to detect anomalies and identify component or module failures.	List of failed modules	Success: 90% Time: 9.1 s
Decision Making	Based on network state [model instance] and performance analysis outcomes [P_e_/P_out_ trends], select the corresponding action from the list: 1) increase transmission power 2) adjust azimuth angle 3) turn on additional module 4) update configuration	Selected action	Success: 95% Time: 6.3 s
Command Generation	Platform-specific control commands are generated according to the selected actions: [actions] and the network elements described in the model instance: [model instance].	Executable platform commands	Success: 85% Time: 10.4 s

## Data Availability

Data and additional resources can be obtained on demand from the corresponding author.

## Abbreviations

The following abbreviations are used in this manuscript:

ABER	Average Bit Error Rate
AF	Amplify-and-Forward
BPSK	Binary Phase Shift Keying
CDF	Cumulative Distribution Function
DF	Decode-and-Forward
DRL	Deep Reinforcement Learning
EMF	Eclipse Modeling Framework
FL	Federated Learning
GenAI	Generative Artificial Intelligence
IoT	Internet of Things
ISAC	Integrated Sensing and Communication
LoS	Line-of-Sight
LLM	Large Language Model
MDE	Model-Driven Engineering
NETCONF	Network Configuration Protocol
NLoS	Non-Line-of-Sight
OCL	Object Constraint Language
PDF	Probability Density Function
P_e_	Average Bit Error Probability
P_out_	Outage Probability
RIS	Reconfigurable Intelligent Surface
R-D	Relay–Destination
S-R	Source–Relay
SNR	Signal-to-Noise Ratio
QoS	Quality of Service
QPSK	Quadrature Phase Shift Keying
RAG	Retrieval Augmented Generation
RPC	Remote Procedure Call
UML	Unified Modeling Language
VLM	Vision–Language Model
XMI	XML Metadata Interchange
XML	Extensible Markup Language

## Appendix A

### Appendix A.1

Generated NETCONF for base station transmission power modification.

<rpc message-id=“101”

xmlns=“urn:ietf:params:xml:ns:netconf:base:1.0”>

<edit-config>

<target>

<running/>

</target>

<config>

<base-stations xmlns=“urn:example:base-station”>

<base-station>

<id>BS_01</id>

<radio>

<transmission-power unit=“dB”>

<increase>2</increase>

</transmission-power>

</radio>

</base-station>

</base-stations>

</config>

</edit-config>

</rpc>

### Appendix A.2

Generated NETCONF payload for azimuth adjustment.

<rpc message-id=“102”

xmlns=“urn:ietf:params:xml:ns:netconf:base:1.0”>

<edit-config>

<target>

<running/>

</target>

<config>

<base-stations xmlns=“urn:example:base-station”>

<base-station>

<id>BS_01</id>

<antenna>

<azimuth unit=“degree”>35</azimuth>

</antenna>

</base-station>

</base-stations>

</config>

</edit-config>

</rpc>

### Appendix A.3

Generated NETCONF payload for activation of backup module.

<rpc message-id=“103”

xmlns=“urn:ietf:params:xml:ns:netconf:base:1.0”>

<edit-config>

<target>

<running/>

</target>

<config>

<base-stations xmlns=“urn:example:base-station”>

<base-station>

<id>BS_02</id>

<operational-state>active</operational-state>

<role>backup</role>

<power-state>on</power-state>

</base-station>

</base-stations>

</config>

</edit-config>

</rpc>
